# Cytosolic malate dehydrogenase 4 modulates cellular energetics and storage reserve accumulation in maize endosperm

**DOI:** 10.1111/pbi.13416

**Published:** 2020-06-14

**Authors:** Yongqiang Chen, Zhiyuan Fu, Hui Zhang, Runmiao Tian, Huili Yang, Canran Sun, Lulin Wang, Wen Zhang, Zhanyong Guo, Xuehai Zhang, Jihua Tang

**Affiliations:** ^1^ National Key Laboratory of Wheat and Maize Crops Science/Collaborative Innovation Center of Henan Grain Crops/College of Agronomy Henan Agricultural University Zhengzhou China

**Keywords:** Maize, cytosolic malate dehydrogenase 4, energetics, starch, zein, mitochondrion

## Abstract

Cytosolic malate dehydrogenase (MDH) is a key enzyme that regulates the interconversion between malate and oxaloacetate (OAA). However, its role in modulating storage compound accumulation in maize endosperm is largely unknown. Here, we characterized a novel naturally occurring maize *mdh4‐1* mutant, which produces small, opaque kernels and exhibits reduced starch but enhanced lysine content. Map‐based cloning, functional complementation and allelism analyses identified *ZmMdh4* as the causal gene. Enzymatic assays demonstrated that ZmMDH4 predominantly catalyses the conversion from OAA to malate. In comparison, the activity of the mutant enzyme, which lacks one glutamic acid (Glu), was completed abolished, demonstrating that the Glu residue was essential for ZmMDH4 function. Knocking down *ZmMdh4 in vivo* led to a substantial metabolic shift towards glycolysis and a dramatic disruption in the activity of the mitochondrial complex I, which was correlated with transcriptomic alterations. Taken together, these results demonstrate that *ZmMdh4* regulates the balance between mitochondrial respiration and glycolysis, ATP production and endosperm development, through a yet unknown feedback regulatory mechanism in mitochondria.

## Introduction

Owing to its high value as animal feed, raw industrial material, food for human consumption and fuel through bioethanol production (Ranum *et al*., 2014), maize (*Zea mays* L.) is widely distributed and cultivated throughout the world. Maize endosperm accounts for over 75% of the kernel dry weight and mainly contains starch and protein. Maize *starchy* endosperm mutants often exhibit changes in the structure and/or accumulation of starch granules and/or protein bodies (PBs), thus affecting grain yield and nutritional quality. Examples include the *opaque* (*o*) and *floury* (*fl*) mutants. *o1*, *o10*, *fl1* and *fl4* mutants have abnormal PBs and noticeable changes in the levels of zeins and alcohol‐soluble prolamins, which are the main components of PBs (Holding *et al*., 2007; Larkins and Hurkman, 1978; Lending and Larkins, 1989; Wang *et al*., 2014a; Wang *et al*., 2012; Yao *et al*., 2016). Several other *opaque/floury* mutants, such as *o2*, *o6*, *o7*, *o11*, *fl2*, *fl3*, *Mucronate*, De*‐B30, *Mto140* and *ocd1*, display reduced zeins and a compensatory increase in non‐zeins, which result in elevated lysine content (Coleman *et al*., 1997; Feng *et al*., 2018; Holding *et al*., 2010; Kim *et al*., 2006; Kim *et al*., 2004; Li *et al*., 2017; Schmidt *et al*., 1992; Wang *et al*., 2011; Wang *et al*., 2014b; Yang *et al*., 2018). In case of *o5*, however, the *opaque* phenotype is caused by morphological alterations in starch granules rather than changes in protein and/or amino acid composition (Hunter *et al*., 2002; Myers *et al*., 2011). These alterations are due to mutations in the structural or regulatory protein genes of zeins. *opaque/floury* mutants, such as *o2*, *o11*, *fl2*, *fl3*, *Mucronate* and De^*^‐B30, involve genes that regulate zein biosynthesis (Coleman *et al*., 1997; Feng *et al*., 2018; Kim *et al*., 2006; Kim *et al*., 2004; Li *et al*., 2017; Schmidt *et al*., 1992); whereas, other *opaque/floury* mutants, such as *o1*, *o10*, *fl1* and *fl4*, are controlled by genes that participate in the assembly of zeins into PBs (Holding *et al*., 2007; Wang *et al*., 2014a; Wang *et al*., 2012; Yao *et al*., 2016). In addition, the *o6*, *o7*, *Mto140* and *ocd1* mutants are impaired in amino acid metabolism or other primary metabolic pathways (Holding *et al*., 2010; Wang *et al*., 2011; Yang *et al*., 2018). For example, *O6* (also known as *Pro1*) and *Mto140* encode Δ^1^‐pyrroline‐5‐carboxylate synthetase and arogenate dehydrogenase 1 that catalyse proline and tyrosine biosynthesis, respectively. Mutations of these two genes resulted in restricted proline and tyrosine supply for zein biosynthesis (Holding *et al*., 2010; Wang *et al*., 2014b). *O7* and *Ocd1*, however, are oxalyl‐CoA synthetase and oxalyl‐CoA decarboxylase genes that are involved in oxalate degradation. These mutations affect amino acid levels and zein accumulation (Wang *et al*., 2011; Yang *et al*., 2018) through an unknown mechanism. Together, these published data suggest a link between amino acid metabolism, zein biosynthesis and endosperm texture. Thus, the cloning and characterization of additional kernel mutants will deepen our understanding of how protein and/or starch biosynthesis are regulated in maize endosperm.

To this end, we isolated a *starchy kernel* mutant with altered starch, zein and lysine content in the endosperm. The mutant phenotype was found to be the result of a 3‐bp deletion in the cytosolic *NAD‐dependent malate dehydrogenase* 4 (*Mdh4*) gene. MDHs are oxidoreductases that catalyse the reversible interconversion between malate and OAA in the cytosol and other organelles, including glyoxysomes, mitochondria, peroxisomes and chloroplasts. In *Arabidopsis thaliana*, the plastidial NAD‐specific MDH shuttles malate and OAA in non‐photosynthetic tissues and its null mutant is embryo lethal (Beeler *et al*., 2014; Selinski *et al*., 2014). In contrast, NADP‐MDH is important for adjusting the ATP/NADPH ratio in light malate valve and loss of NADP‐MDH has no or little effect on growth (Hebbelmann *et al*., 2012; Heyno *et al*., 2014). Overexpressing maize NADP‐dependent MDH in *A*. *thaliana* confers salt tolerance (Kandoi *et al*., 2018). Mitochondrial NAD‐MDHs play a critical role in the tricarboxylic acid (TCA) cycle and null mutants of the two NAD‐MDH isoforms display viable but abnormal plant development (Sew *et al*., 2016; Tomaz *et al*., 2010). Peroxisomal NAD‐MDHs mainly generate NAD^+^ for the β‐oxidation of fatty acids and double mutant of the two isoforms fail to efficiently mobilize triacylglycerols (Pracharoenwattana *et al*., 2007, 2010). Although loss‐of‐function mutants of the plastidial, mitochondrial and peroxisomal MDH isoforms have been characterized, the phenotypic expression of cytosolic MDH of *A*. *thaliana* mutants is largely unknown. Cytosolic MDH mediates malate biosynthesis in the cytosol and has been reported to participate in plant and cell growth in apple (Wang *et al*., 2016; Yao *et al*., 2011a, 2011b). Its overexpression confers abiotic stress tolerance, such as cold and salt exposure, in apple trees by modulating redox homeostasis via malate accumulation (Wang *et al*., 2016; Yao *et al*., 2011a, 2011b). Recently, a NAD‐dependent cytosolic MDH (*flo16*) has been reported in rice, revealing a potential role of cytosolic MDHs on reserve storage and seed development in the grass family (Teng *et al*., 2019). The mutant exhibits reduced ATP production and enhanced oxidation via a reduction in the activities of starch biosynthetic enzymes, leading to decreased starch accumulation in rice endosperm. Conversely, the overexpression of cytosolic MDH significantly improves grain weight (Teng *et al*., 2019). Taken together, these data point to a role of cytosolic MDHs in regulating starch biosynthesis and seed development in grass species.

The maize genome contains four NAD‐dependent MDH isoforms and one NADP‐dependent MDH isoform (Goodman *et al*., 1981). However, data concerning the roles of these isoforms are limited. Of these, *ZmMdh1* (Zm00001d009640), *ZmMdh2* (Zm00001d039089) and *ZmMdh3* (Zm00001d044042) are localized to mitochondria, *ZmMdh4* (Zm00001d032695) and *ZmMdh5* (Zm00001d014030) to the cytosol, and *ZmMdh6* (Zm00001d031899) to plastids (Goodman *et al*., 1981; Newton and Schwartz, 1980). The cytosolic isoforms *ZmMdh4* and *ZmMdh5* are a result of gene duplication and reside on different chromosomes (Mcmillin and Scandalios, 1980). In maize, at least one of the two mitochondrial MDHs needs to be present to ensure normal kernel development (Goodman *et al*., 1981), suggesting that MDH activity is indispensable for maize kernel and plant development under non‐stress conditions. By contrast, the null homozygous mutants of either or both cytosolic MDHs are viable and can grow to maturity (Goodman *et al*., 1981), suggesting a potential regulatory role in endosperm development and storage reserve accumulation.

In this study, we isolated the maize kernel mutant, *mdh4‐1*, which shows a deformed endosperm, reduced starch and zein content, and enhanced lysine content. Map‐based cloning, genetic transformation and allelism analyses confirmed cytosolic *ZmMdh4* as the causal gene. A 3 base pairs (bps) deletion in exon 7 of *ZmMdh4* was validated as the functional mutational site. This mutation renders the ZmMDH4 enzyme almost inactive for converting OAA to malate, likely by altering the tertiary structure of the enzyme. The mutant shows elevated *Zmmdh4* transcript and protein levels, presumably to compensate for the reduction in enzymatic activity. Combined with the transcriptome and metabolome data of immature kernels at 12 days after pollination (DAP), we propose a regulatory model of *ZmMdh4* on storage reserve accumulation in maize.

## Results

### A novel naturally occurring *mdh4‐1* mutant displays small and opaque kernels, reduced starch content and elevated lysine levels

**Figure 1:**
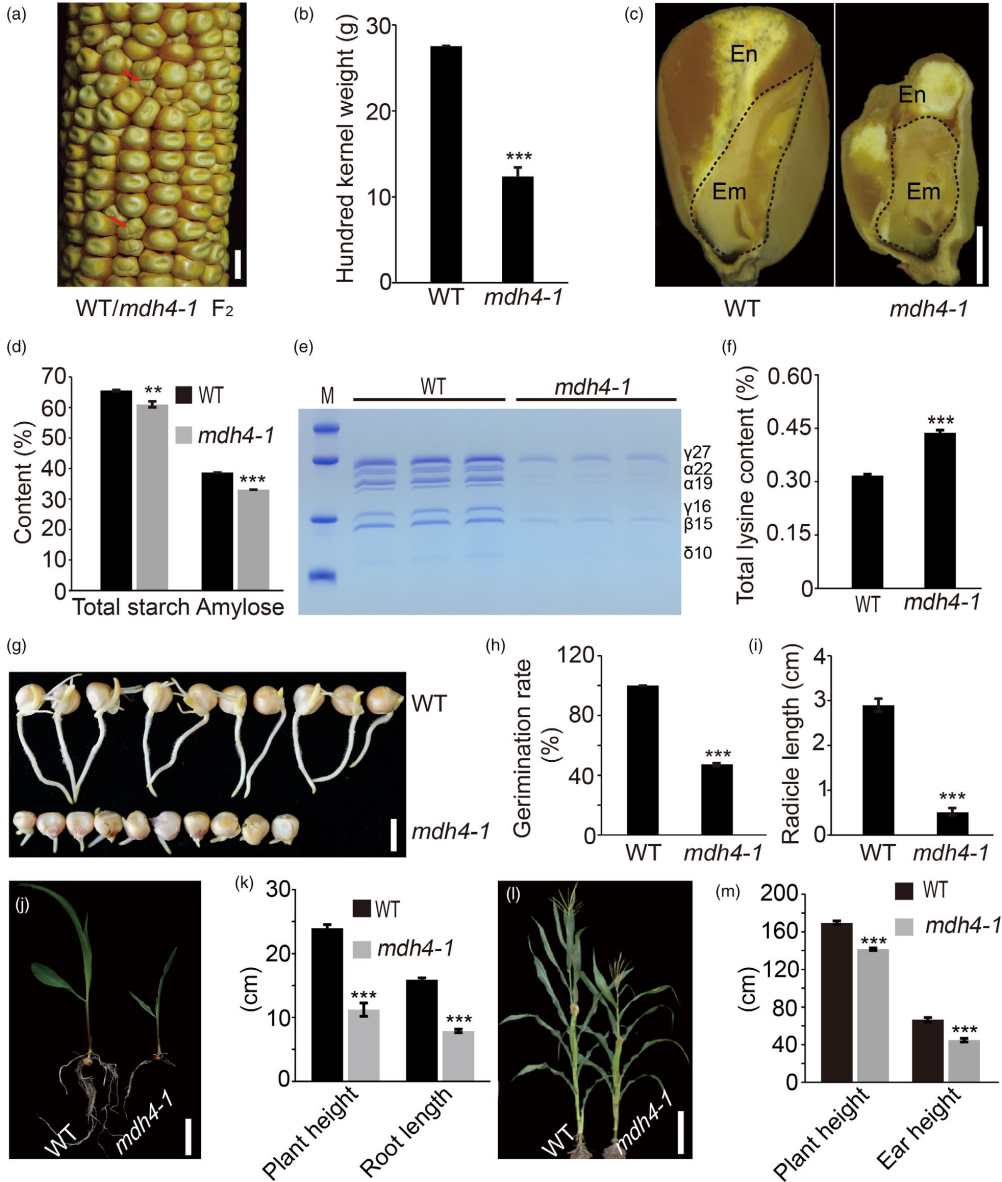
Phenotypic comparison between WT and the *mdh4‐1* mutant. (a) Segregation of Zheng58 × *mdh4‐1* F_2_ seeds. The red arrows indicate *mdh4‐1* kernels; scale bar, 1 cm. (b) 100‐kernel weight of the WT *vs*. *mdh4‐1,* with five replicates. (c) Comparison between the longitudinal sections of WT and *mdh4‐1* kernels. En, endosperm; Em, embryo; scale bar 2 mm. (d) Starch and amylose content in WT *vs*. *mdh4‐1* endosperms with four replicates. (e) Zein proteins in 12 DAP endosperms of WT and *mdh4‐1*. (f) Total lysine content in WT *vs*. *mdh4‐1* mature kernels with three replicates. (g) Representative examples of germination of the WT and *mdh4‐1* seeds; scale bar, 1 cm. (h) Germination rate of WT *vs*. *mdh4‐1* with three replicates. (i) Radical length of WT *vs*.*mdh4‐1* seedlings with three replicates. (j) Representative examples of WT and *mdh4‐1* seedlings 13 days after germination (DAG); scale bar, 5 cm. (k) Height and root length of WT vs. *mdh4‐1* seedlings 13 DAG, with ten replicates. (l) Representative examples of mature WT and *mdh4‐1* plants; scale bar, 30 cm. (m) Plant height and ear length of mature WT *vs*. *mdh4‐1* plants, with 15 replicates. In all bar graphs, values are represented as means ± SE, **P < 0.01, ***P < 0.001 denote statistically significant differences between WT and *mdh4‐1* (Student's *t*‐test).

*mdh4‐1* was originally isolated as a small kernel mutant with a deformed endosperm crown (Figure 1a), which appeared 12 DAP on segregating F_2_ ears. These mutants were characterized by the relatively smaller kernel size and delayed development (Figure S1). At maturity, the 100‐kernel weight of homozygous mutant kernels was only 55.1% of the wild type (WT), and the length, width and thickness were 10.8%, 16.5% and 29.8%, lower than those of the WT, respectively (*P* < 0.001, Student's *t*‐test; Figure 1b and Figure S2a‐b). In addition, the longitudinal sections of the kernels showed that the mutant embryo was abnormal and smaller in size compared with that of the WT (Figure 1c). Upon quantification of the storage constituents, a 6.9% and 14.5% reduction in total starch and amylose content, respectively, was observed in mature mutant kernels as compared to the WT (Figure 1d). Furthermore, 12 DAP *mdh4‐1* endosperms accumulated a much lower level of zein protein than WT (Figure 1e). At maturity, the mutant endosperms had an increased level of non‐zein proteins (Figure S2c‐d), which was accompanied by an increase in lysine, one of the major components of non‐zeins, by approximately 1.4‐fold (Figure 1f, Table S2).

In addition to the kernel phenotypes, the *mdh4‐1* mutant also exhibited various growth defects. For example, it exhibited a 50% lower germination rate than the WT (Figure 1h), and radicle root length of the mutant was much shorter than that of the WT (Figure 1g, i). Moreover, the mutant seedlings produced less and shorter roots than WT seedlings at 13 days after germination (DAG) (Figure 1j‐k). Lastly, mutant plant height and ear length were also reduced in comparison to the WT (Figure 1l‐m). These observations suggest that the *mdh4‐1* mutation exerts pleiotropic effects on plant growth and kernel development.

### The *mdh4‐1* mutant exhibits delayed endosperm and embryo development

**Figure 2:**
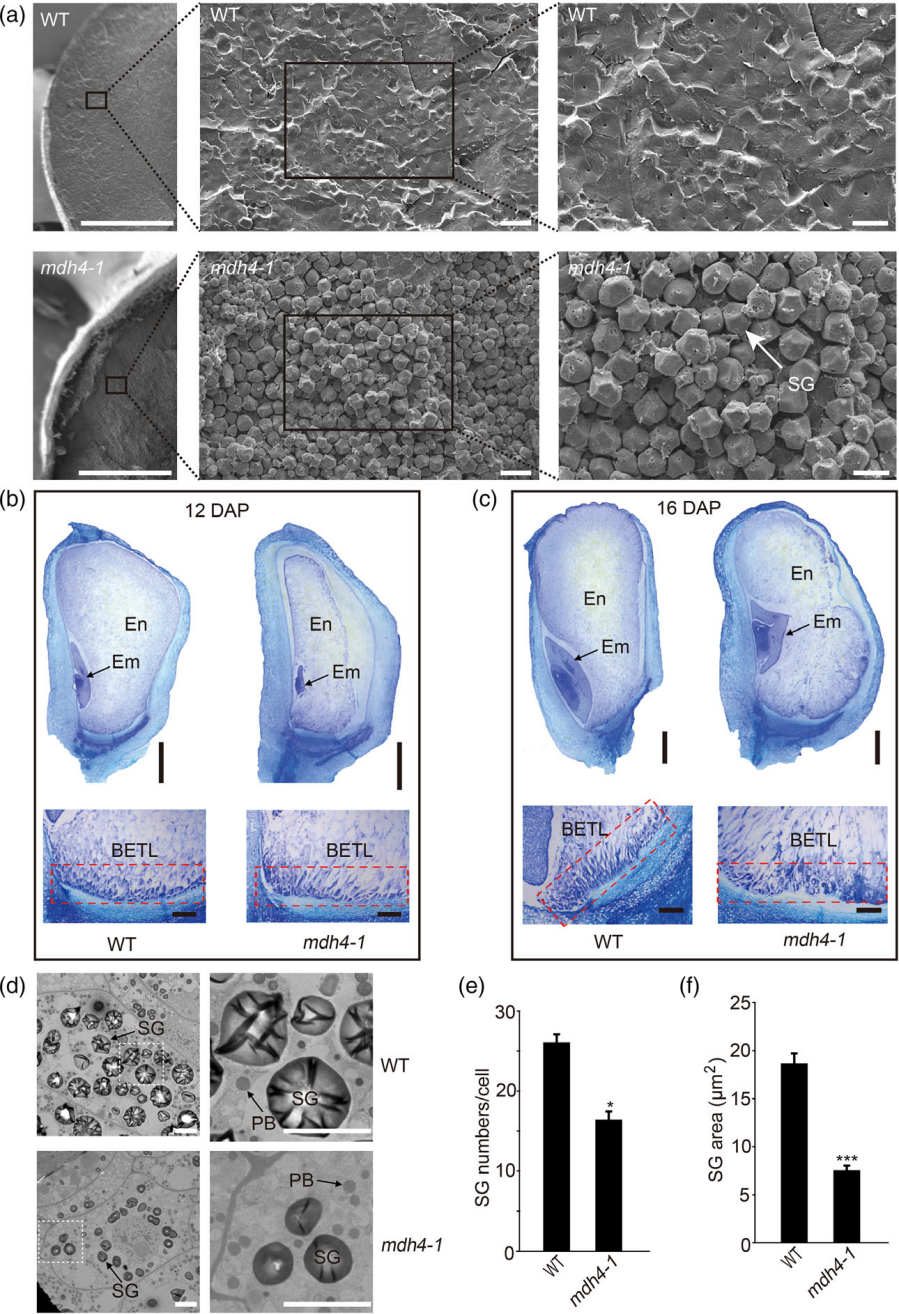
Cytological characterization of WT and *mdh4‐1* seeds at different stages. (a) Scanning electron microscopy (SEM) images of mature WT and *mdh4* endosperms; scale bars, 10 µm. (b) Paraffin sections of 12 DAP and (c) 16 DAP of WT and *mdh4‐1* kernels. Scale bars of whole seed and basal endosperm transfer layer (BETL, red rectangle), 1 mm and 200 µm, respectively. En, endosperm; Em, embryo. (d) Transmission electron microscopy (TEM) images of WT and the *mdh4‐1* mutant seeds 18 DAP. Scale bars in left and right panels, 5 µm and 1 µm, respectively. (e) Number and (f) size of starch grains in WT *vs*. *mdh4‐1* endosperms at 18 DAP with eight replicates. En, endosperm; Em, embryo; SG, starch granule; PB, protein body; M, mitochondria. Values are represented as means ± SE, **P < 0.01, ***P < 0.001 (Student's *t‐*test).

To further dissect the starchy‐like kernel phenotype, sections of mature and developing *mdh4‐1* and WT kernels were examined. Under a scanning electron microscope (SEM), mature mutant kernels displayed irregularly shaped starch granules with reduced surrounding matrix, whereas the starch granules in mature WT kernels aggregated together and were surrounded by a dense matrix (Figure 2a). The paraffin sections showed that *mdh4‐1* embryos developed much more slowly than those of the WT. Notably, the shoot and root apical meristems, and leaf primordia were clearly visible in the WT embryos, but were still indistinguishable in mutant embryos at 12 DAP (Figure 2b). Additionally, starchy endosperm development was severely retarded in the mutant as compared with the WT, with the starchy endosperm occupying only 2/3 of the kernel as to 100% occupancy in the WT (Figure 2c). At 16 DAP, the mutant embryo was deformed and the endosperm had developed into three blocks (Figure 2c). These observations support the notion that embryo and endosperm development were delayed in the *mdh4‐1* mutant. In addition, reduced ingrowth in the cells of the basal endosperm transfer layer (BETL) in *mdh4‐1* kernels as compared to the WT kernels was observed, suggesting impaired transmission of nutritional constituents from the maternal tissue to endosperm cells (Figure 2b‐c).

The starchy endosperm cells were examined by transmission electron microscopy (TEM), and it was found that the shape and number of PBs were similar between 18 DAP WT and mutant endosperms (Figure 2d). However, *mdh4‐1* kernels exhibited smaller starch granules than the WT (Figure 2e‐f), which may be related to the loosely packed starch granules and reduced 100‐kernel weight of the mutant kernels (Figures 1b and 2a).

### Map‐based cloning of *mdh4‐1*

For genetic analysis, *mdh4‐1* was crossed with a widely used Chinese elite inbred line, Zheng58, and the resulting F_1_ progeny was self‐pollinated to generate an F_2_ population (Figure 1a). The ratio of WT to mutant F_2_ kernels was roughly 3:1, suggesting the existence of a single recessive mutation. We randomly selected and grew F_2_ kernels that showed the WT phenotype and collected and analysed 100 F_3_ ears. The ratio of non‐segregating (*n* = 30, homozygote) to segregating ears (*n* = 70, heterozygote) was approximately 1:2 (*χ* ^2^ = 0.5 < $\chi_{0.05}^{2}$ = 3.84). In addition, the segregation of normal to mutant F_3_ kernels obtained from segregating F_2_ ears followed a 3:1 ratio (normal:*mdh4‐1* = 5494:1833, *χ* ^2^ = 0.002 < $\chi_{0.05}^{2}$ = 3.84; Table S1). Similar segregation ratios were observed in the other five F_2_ segregating populations constructed by crossing *mdh4‐1* with different inbred lines (Table S1 and Figure S2e). Collectively, these results indicate that the mutant phenotype of *mdh4‐1* is caused by a single recessive locus.

**Figure 3:**
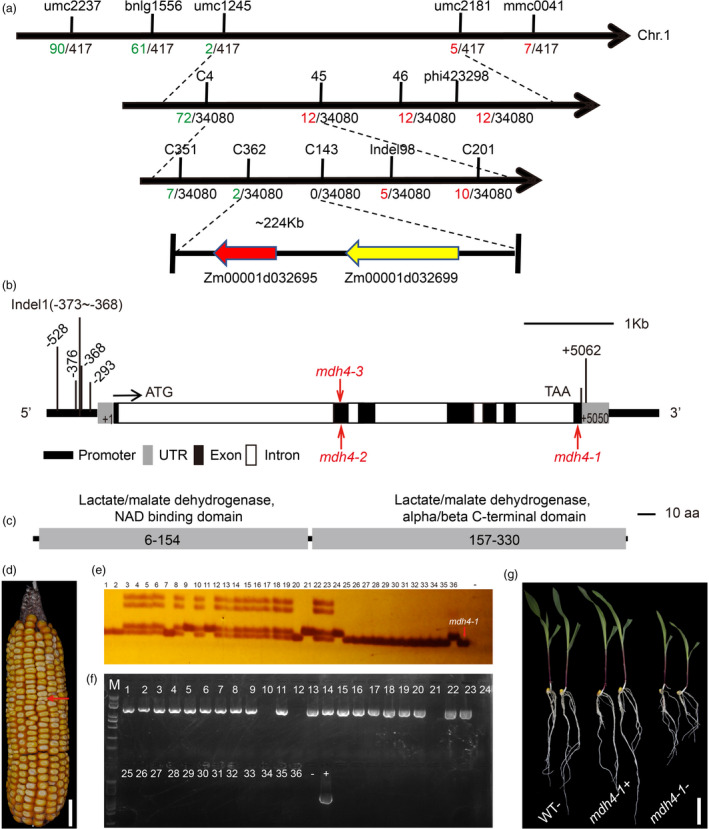
Mapping and genotyping of *ZmMdh4*. (a) Fine mapping of *ZmMdh4* using the F_2_ populations derived from Zheng58 and *mdh4‐1*. The *ZmMdh4* locus was mapped to a ~224 kb region on chromosome 1. The number of recombinants and the population size are shown on the left and right of each marker, respectively. (b) The gene structure of *ZmMdh4*. Allelic mutants are indicated by red letters. The black and white boxes indicate exons and introns, respectively. The bold black line indicates the promoter and the gray boxes indicate the untranslated regions (UTRs). (c) A schematic diagram of the ZmMDH4 protein, with conserved domains indicated. (d) *Mdh4‐OE*/*Mdh4* × *mdh4‐1* F_2_ ear. *Mdh4‐OE* denotes the *Mdh4* overexpression line. The red arrow indicates a *mdh4‐1* mutant kernel. Scale bar, 2 cm. (e‐f) Genotyping of randomly selected kernels. (e) Marker Exon7‐L/Exon7‐R for identifying homozygous *ZmMdh4*. (f) Marker CUB‐R/CUB‐F for identifying positive *ZmMdh4*‐overexpressing individuals. Lanes 1‐24 and 25‐35 show the genotyping results of the normal and mutant kernels, respectively. Lane 36 shows the ZZC01 control. “‐” and “+” represent the blank (H_2_O) and the CUB‐*ZmMdh4* vector control, respectively. M represents the marker. (g) Differences in the phenotypic expression of *Mdh4*‐*OE* lines at ten‐day after germination (DAG). WT‐, non‐transgenic lines with *ZmMdh4* gene; *mdh4*+, positive transgenic lines with *ZmMdh4* gene; *mdh4‐,* negative transgenic lines with *ZmMdh4* gene. The scale bar, 5 cm.

Preliminary genetic mapping using 419 F_2_ individuals placed the target gene between the simple sequence repeat (SSR) markers umc1245 and umc2181 (Table S3) on bin1.08 of chromosome 1. The interval was further narrowed down to a ~224‐kb region between markers C362 (two recombinants) and Indel‐98 (five recombinants) using an F_2_ population consisting of 34,080 individuals (Figure 3a). The 224‐kb target region contains two predicted open reading frames (ORFs), *Zm00001d032695* and *Zm00001d032699*, in the B73 reference genome (RefGen V4; Figure 3a). The genomic sequences of these two genes were compared between *mdh4‐1* and Zheng58 and several single nucleotide polymorphisms (SNPs) were identified, as well as insertions and deletions (InDel). However, only the 3‐bp deletion in exon 7 of *Zm00001d032695* was found exclusively in the *mdh4‐1* mutant (Figure 3b; Figure S3). *Zm00001d032695* is annotated as a cytosolic malate dehydrogenase 4 in *Gramene* (http://www.gramene.org/Zea_mays/) and was therefore named *ZmMdh4*. *ZmMdh4* is ~5.4‐kb in length with seven exons (Figure 3b), and the full‐length cDNA of *ZmMdh4* is estimated to be 1381‐bp. The deduced protein translation contains 332 amino acids, with a molecular mass of ~36 kD. It has a predicted NAD‐dependent cytoplasmic malate dehydrogenase domain (Figure 3c). The polymorphic sites of *ZmMdh4* were analysed in an association panel consisting of 540 inbred lines to identify the functional polymorphisms associated with kernel characteristics (http://www.maizego.org/Resources.html; Table S4). Three SNPs in the 5' UTR were found to associate with lysine content and/or kernel thickness (Figure 3b and Table S5). Based on these results, *ZmMdh4* was selected as the candidate gene for further validation and characterization.

To validate whether *ZmMdh4* is the causal genetic basis for the observed *mdh4‐1* kernel phenotype, the *ZmMdh4* coding sequence was overexpressed using the constitutive ubiquitin (Ubi) promoter. The resulting transgenic line (*Mdh4‐OE*) was crossed with a homozygous *mdh4‐1* (−/−) mutant, and F_1_ was self‐pollinated to generate F_2_ seeds (Figure 3d). Twenty‐four normal (WT) and 11 mutant F_2_ kernels were selected randomly and genotyped with markers detecting the 3‐bp deletion in *mdh4‐1* (−/−) and the *Mdh4‐OE* transgene. Four of the 24 normal kernels had the homozygous *mdh4‐1* (−/−) genotype with *Mdh4‐OE* (Figure 3e‐f). Additionally, they showed phenotypic characteristics analogous to those of the normal kernels at the seedling stage (Figure 3g). These results indicate that *Mdh4‐OE* could rescue the *mdh4‐1* mutant phenotype. Together, these data confirmed that *ZmMdh4* was the causal gene of the *mdh4‐1* mutant.

### Knocking out *ZmMdh4*, but not *ZmMdh5*, phenocopies *mdh4‐1* phenotypes

**Figure 4:**
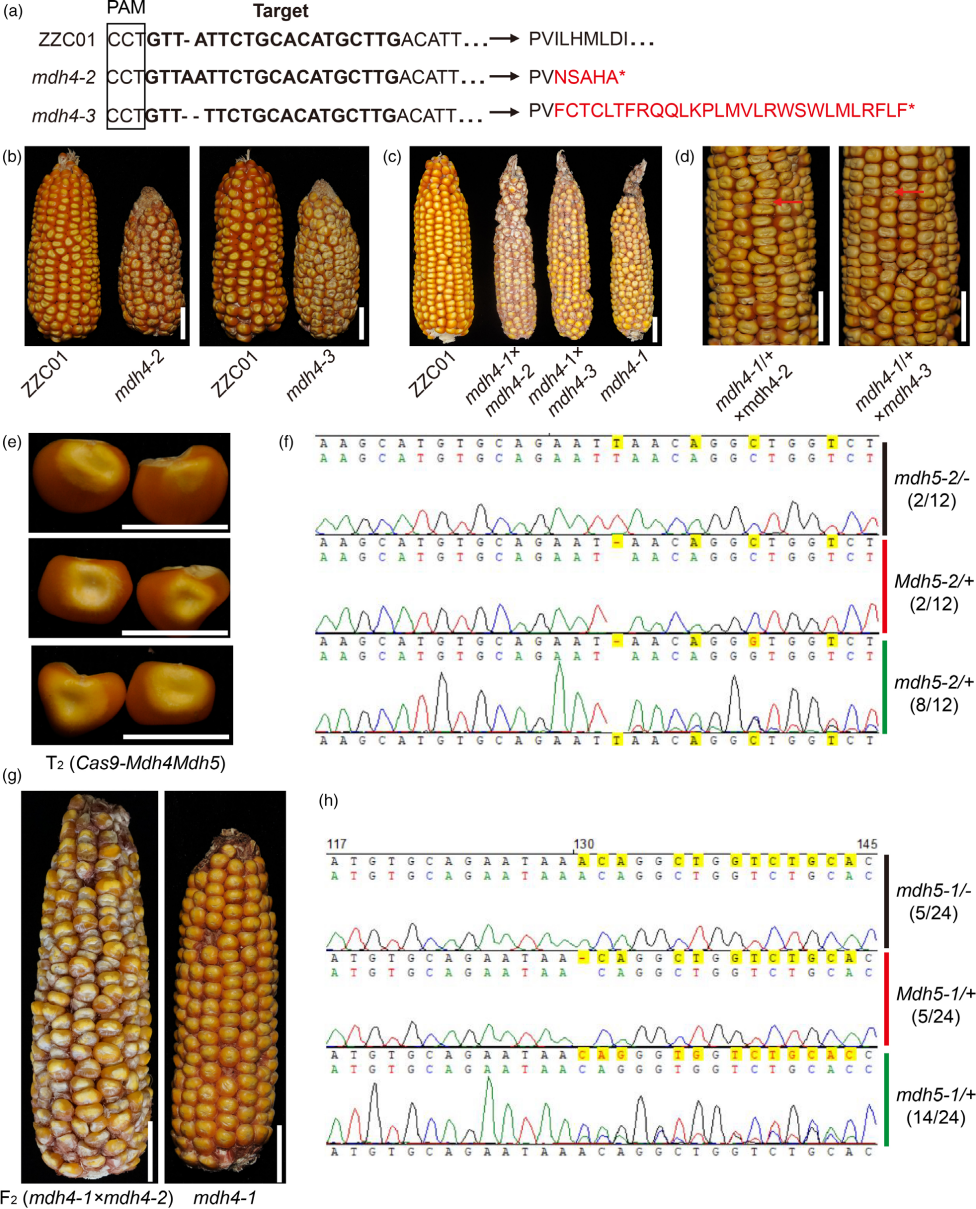
Validation of the *ZmMdh4* transgenic lines. (a) Alignment of the genomic and amino acid sequences of the positive Cas9‐edited transgenic plants and the control ZZC01. The bold letters in black denote the sgRNA target sequence. (b) Comparison of the *mdh4‐2* and *mdh4‐3* ears with ZZC01. Scale bars, 2 cm. (c) Allelism test using homozygous *mdh4‐1*, *mdh4‐2*, and *mdh4‐3*. The scale bars are 2 cm. (d) Allelism test using heterozygous *mdh4‐1* and homozygous *mdh4‐2* and *mdh4‐3* mutants. The scale bars, 2 cm. The red arrows indicate mutant kernels. (e) Representative kernels from T_2_ Cas9‐*Mdh4* (+/‐) *Mdh5* (+/‐); scale bars, 1 cm, (f) Linkage analysis of *Zmmdh5* with randomly selected normal kernels from the *mdh4‐2* T_2_ generation. (g) Representative *mdh4‐1* × *mdh4‐2* and *mdh4‐1* F_2_ ears; scale bars, 2 cm. (h) Linkage analysis of *ZmMdh5* with randomly selected kernels from the *mdh4‐1* × *mdh4‐2* F_2_ ear.

To further confirm *ZmMdh4* as the causal gene of the *mdh4‐1* mutant phenotype, Mu‐inserted *ZmMdh4* mutants were screened from the Maize Genetics Stock Center. However, the only *Mu*‐mutant identified had the *Mu* insertion in the non‐coding region of *ZmMdh4*, which exhibited no obvious kernel phenotype. Thus, CRISPR/Cas9 was used to generate *ZmMdh4* loss‐of‐function lines with a specific guide RNA (gRNA) using ZZC01. Sequencing of the CRISPR/cas9‐mediated *ZmMdh4*‐edited transgenic individuals resulted in the identification of two mutants, *mdh4‐2* and *mdh4‐3*, that carry null mutations in the *ZmMdh4* gene (Figure 4a). The T_1_ individuals of *mdh4‐2* and *mdh4‐3* were self‐pollinated for two consecutive generations to produce homozygous T_3_ progeny (Figure 4b). Allelism tests were performed by crossing the homozygous *mdh4‐2* (−/−) and *mdh4‐3* (−/−) T_3_ plants with both the homozygous *mdh4‐1* (−/−) and heterozygous (+/−) individuals, respectively. The results show that the F_2_ kernels of the *mdh4‐2* (−/−) *× mdh4‐1* (−/−) cross all phenocopied homozygous *mdh4‐1* (−/−) kernels (Figure 4c), and the *mdh4‐2* (−/−) *×* (+/−) and F_2_ kernels exhibited a mutant:WT ratio of 1:1 (Figure 4d and Table S6). Similar results were obtained with *mdh4‐3* (−/−) derived F_2_ kernels. These findings confirmed that the CRISPR/cas9‐mediated mutations were allelic to the naturally occurring *mdh4‐1* mutation. In support of this conclusion, kernel size and 100‐kernel weight of the *mdh4‐2* (−/−) and *mdh4‐3* (−/−) T_3_ individuals were found to be comparable to those of the *mdh4‐1* mutant (Figure S4a‐c). In addition, the germination rate, seedling and adult height, as well as root length of the two cas9 lines, were reduced by more than 50% as compared with the control, similar to that observed for *mdh4‐1* (Figure S4d‐i). These results further supported *ZmMdh4* as the target gene.

Due to the high sequence similarity between *ZmMdh5* and *ZmMdh4* (McMillin and Scandalios, 1980, *Gramene*; http://ensembl.gramene.org/Zea_mays), both genes were knocked out by CRISPR/cas9 in *mdh4‐2* and *mdh4‐3*. To test whether the cas9‐mediated mutations in *ZmMdh5* had contributed to the mutant phenotype, *Zmmdh5*‐linked markers were assayed. They did not co‐segregate with kernel phenotype in the T_2_ generation (Figure 4e‐f), suggesting that the *ZmMdh5* mutations were not causal of the mutant kernel phenotype in the two cas9 lines. Further, the F_2_ kernels derived by crossing *mdh4‐1* (*Mdh5* (+/+) *mdh4* (−/−)) with *mdh4‐2* (*Mdh5* (+/−) *mdh4* (−/−)) all exhibited the mutant phenotype (Figure 4g‐h). These results suggest that the *ZmMdh5* cas9 mutations were unrelated to the mutant kernel phenotype, and *ZmMdh4* was the target gene.

### *ZmMdh4* is constitutively expressed and its protein is localized to the cytoplasm

**Figure 5:**
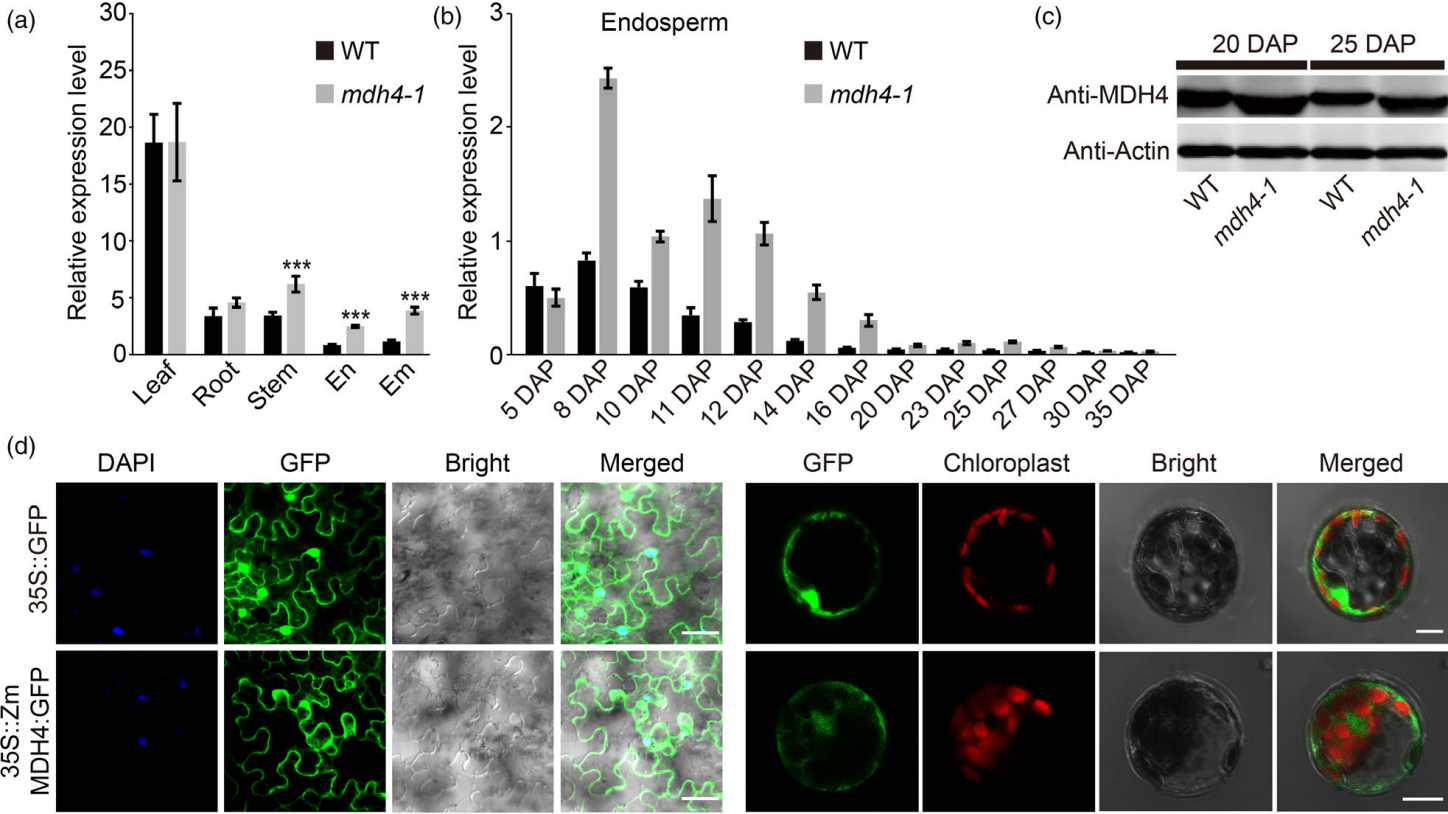
Expression pattern and subcellular localization of *ZmMdh4*. (a) Quantitative PCR analysis of *ZmMdh4* relative transcript abundance in various tissues from WT *vs*. *mdh4‐1* mutant plants. Values are represented as means ± SE of three biological replicates. ^***^ *P* < 0.001, *t*‐test. (b) *ZmMdh4* expression during endosperm development. (c) The abundance of ZmMDH4 protein in the WT *vs*. *mdh4‐1* mutant. (d) Subcellular localization of ZmMDH4 in *Nicotiana benthamiana* leaves and *Arabidopsis* mesophyll protoplasts; scale bars are 100 μm.

Using qPCR, *ZmMdh4* expression was quantified in the root, stem, leaf, embryo, and endosperm of the WT and *mdh4‐1* mutant. *ZmMdh4* was found to be constitutively expressed in all tested tissues, with the highest expression level in the leaf (Figure 5a). During kernel filling, *ZmMdh4* expression was mainly detected during the early stages of endosperm development and its transcript level was higher in the mutant than the WT (Figure 5b). Consistent with this result, Western blot analysis with the MDH4 antibody indicated a relatively higher ZmMDH4 protein level in *mdh4‐1* kernels even during late endosperm development (Figure 5c).

In *Gramene* (http://ensembl.gramene.org/Zea_mays), ZmMDH4 is predicted to be localized in the cytoplasm. To verify this prediction, the full‐length CDS of *ZmMdh4* was C‐terminally fused to the enhanced green fluorescent protein (eGFP) and transiently expressed under the control of the CaMV 35S promoter in *Nicotiana benthamiana* epidermal leaf cells and *A*.* thaliana* mesophyll protoplasts. In both approaches, the fusion protein was found localized in the cytoplasm (Figure 5d), thus confirming the localization of ZmMDH4 to the cytoplasm. On the contrary, free GFP signal was detected in the nuclei and the cytoplasm.

### ZmMDH4 predominantly catalyzes the conversion from OAA to malate

**Figure 6:**
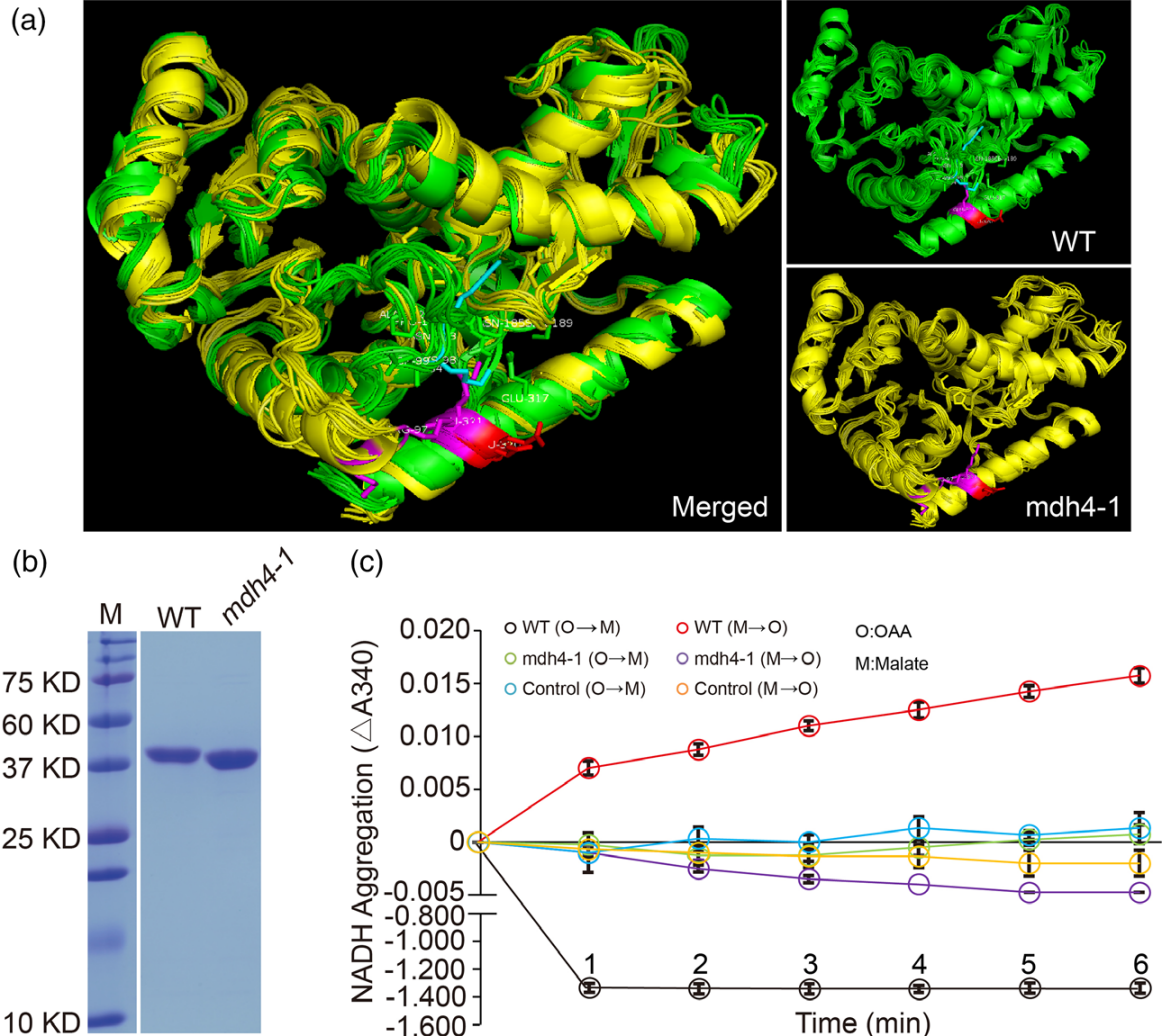
Enzymatic characterization of ZmMDH4. (a) Predicted 3D structure of the WT and mutant ZmMDH4. (b) SDS‐PAGE analysis of the His‐tagged WT and *mdh4‐1* mutant proteins. M, protein standard with molecular weights listed on the left. (c) Determination of WT and mutant ZmMDH4 oxidoreductase activity by detecting the change in NADH levels at 340 nm, with four replicates. A total of 5 μg protein was used for initial loading.

The 3‐bp deletion in the *mdh4‐1* mutant results in a Glu deletion at position 322 in the C‐terminus of the MDH4 protein, which is distant from the active site (located at 156–168aa; Uniprot database, http://www.Uniprot.org). However, this deletion may alter the overall conformational structure of the ZmMDH4 protein. Specifically, Arg97 and Lys98, the spatial neighbours of Glu322, reside in a loop (88‐103aa) associated with substrate binding (SWISS‐MODEL algorithm; Waterhouse *et al*., 2018). The +/− salt bridge between Arg97 and Glu322 draws the loop and helix9 together in the WT MDH4 enzyme. In the mutant enzyme, however, the +/− salt bridge is abolished by the change of Lys98, thereby potentially loosening the MDH4 structure by preventing the interaction between the loop and helix 9 (Figure 6a).

To examine the effect of Glu322 deletion on MDH4 function, purified WT and mutant MDH4 protein heterologously expressed in bacteria were used in enzymatic assays to monitor changes in NADH levels (Figure 6b‐c). Very little change in NADH levels was observed during the malate‐to‐OAA conversion for both enzymes but a substantial decrease in NADH with the WT MDH4 enzyme during the OAA‐to‐malate conversion, suggesting that MDH4 mainly catalyses the reaction from OAA to malate (Figure 6c). Consistent with our hypothesis, the mutant enzyme almost completely lost its activity for catalysing the OAA‐to‐malate conversion, as no obvious change in NADH was observed (Figure 6c). Thus, 3‐bp deletion in exon 7 significantly impacts MDH4 activity presumably by causing a conformational change in MDH4 tertiary structure.

### Disruption of *ZmMdh4* leads to changes in cellular energetics and impairment of mitochondrial complex I and II function

**Figure 7:**
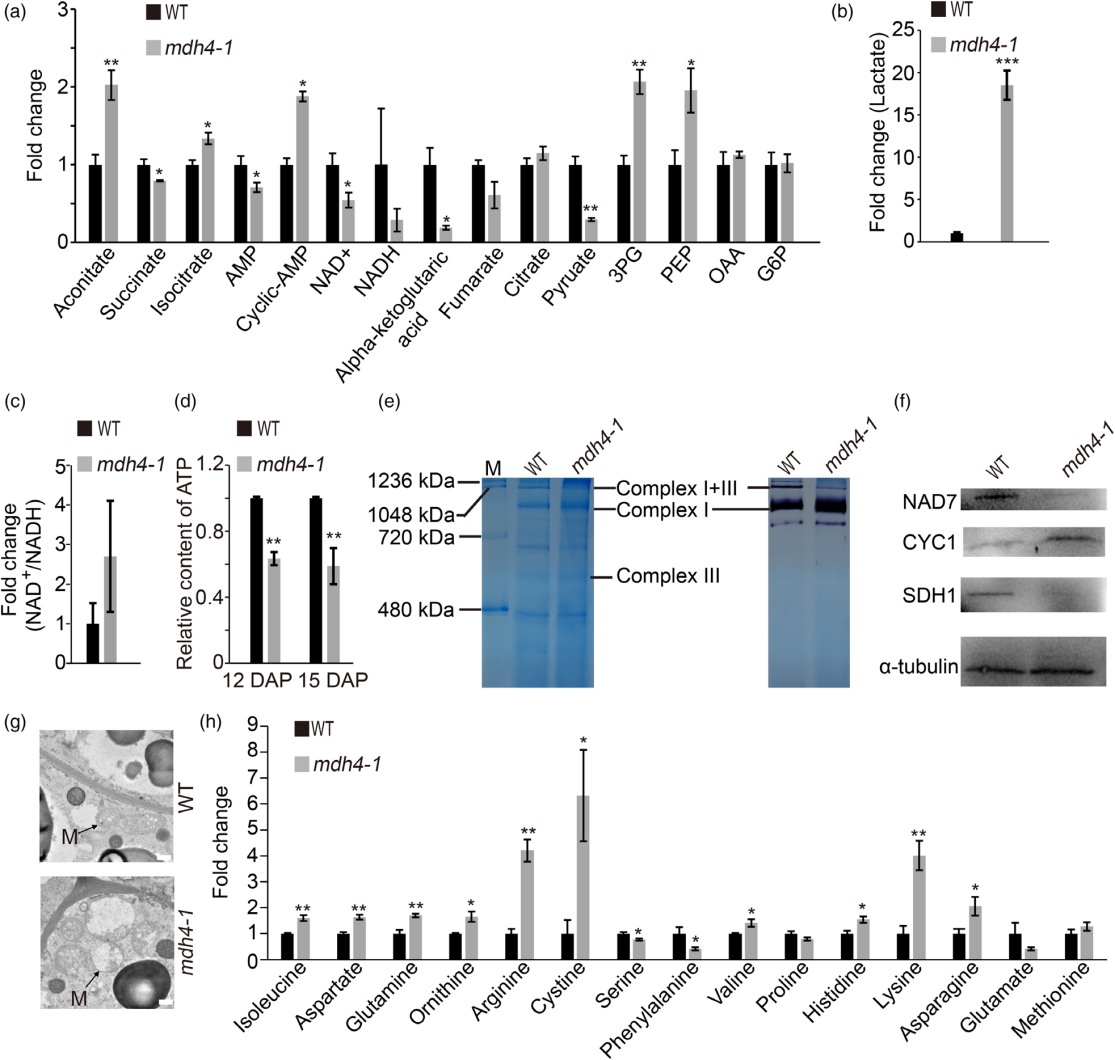
Targeted metabolomic analysis of WT and *mdh4‐1* kernels. (a) Fold change of energy metabolite levels in *mdh4‐1* kernels over WT. (b) Relative content of lactate of *mdh4‐1* as compared to WT. (c) Fold change of the NAD^+^/NADH ratio in *mdh4‐1* as compared to WT. (d) Relative ATP content in the developing endosperm of WT and *mdh4‐1* 12 and 15 DAP, with three replicates. (e) BN‐PAGE assay of mitochondrial complex assembly and in‐gel NADH enzyme activity from immature kernels of the *mdh4‐1* mutant and WT at 15 DAP. M, protein standard. (f) Western blot with NAD7, SDH1, and CYC1 antibodies against protein isolated from WT and *mdh4‐1* kernels. (g) The ultrastructure of mitochondrion (M) in *mdh4‐1* and WT kernel cells; scale bar, 500 nm. (h) Fold differences in overall amino acid content of *mdh4‐1* as compared to WT kernels. Error bars indicate the SE of three biological replicates. *P < 0.05, **P < 0.01, ***P < 0.001 (Student's *t‐*test).

Because the conversion of OAA to malate is a key step in the TCA cycle, the amount of ATP and metabolites associated with energy metabolism in 12 DAP *mdh4‐1* and WT kernels were quantified. Specifically, this was undertaken to investigate if the lack in ZmMDH4 activity affected energy production. The levels of lactate, aconitate, 3‐phosphoglycerate (3PG), phosphoenolpyruvate (PEP) and cyclic‐AMP increased significantly by >1.5‐fold in *mdh4‐1* compared with the WT, whereas those of NAD^+^, NADH, pyruvate and alpha‐ketoglutaric acid (α‐KG acid) were reduced by >1.5‐fold (Figure 7a‐b). These data suggest that both glycolysis and the TCA cycle were altered. By contrast, the ratio of NAD^+^/NADH, which reflects the redox state, increased by 2.7‐fold in the *mdh4‐1* mutant as compared to the WT (Figure 7c). Taken together, these results suggest that the TCA cycle is impaired in the *mdh4‐1* mutant. This is further supported by the observation that ATP content decreased ~40% in the 12 DAP mutant kernels compared with the WT (Figure 7d). Consistently, we observed reduced NADH dehydrogenase activity and the disassociation of the mitochondrial ATP‐producing I + III super‐complex in 15 DAP mutant kernels compared with the WT, as fewer I + III super‐complexes were observed in the mutant than WT (Figure 7e). Further supporting evidence came from the substantial decreases in NAD7 and SDH1 proteins, as well as an increase in CYC1 protein in 15 DAP mutant kernels (Figure 7f). These results are consistent with the vacuolization of the mutant mitochondria in 15 DAP kernels (Figure 7g), suggesting that the disruption of ZmMDH4 led to mitochondria dysfunction and reduced ATP production.

The loss of ZmMDH4 activity in mutant kernels also affected the levels of amino acids derived from the intermediates of the TCA cycle (Figure 7h). For example, we detected the accumulation of amino acids derived from the Asp‐derived pathway and those from the Glu‐derived pathway that competes with Pro, an important component of the zein protein, for the Glu substrate (Figure 7h). These results indicate that the mutant endosperm had developmental defects and alterations in central metabolism.

### Coupling metabolic changes to transcriptomic alterations upon ZmMDH4 depletion

**Figure 8:**
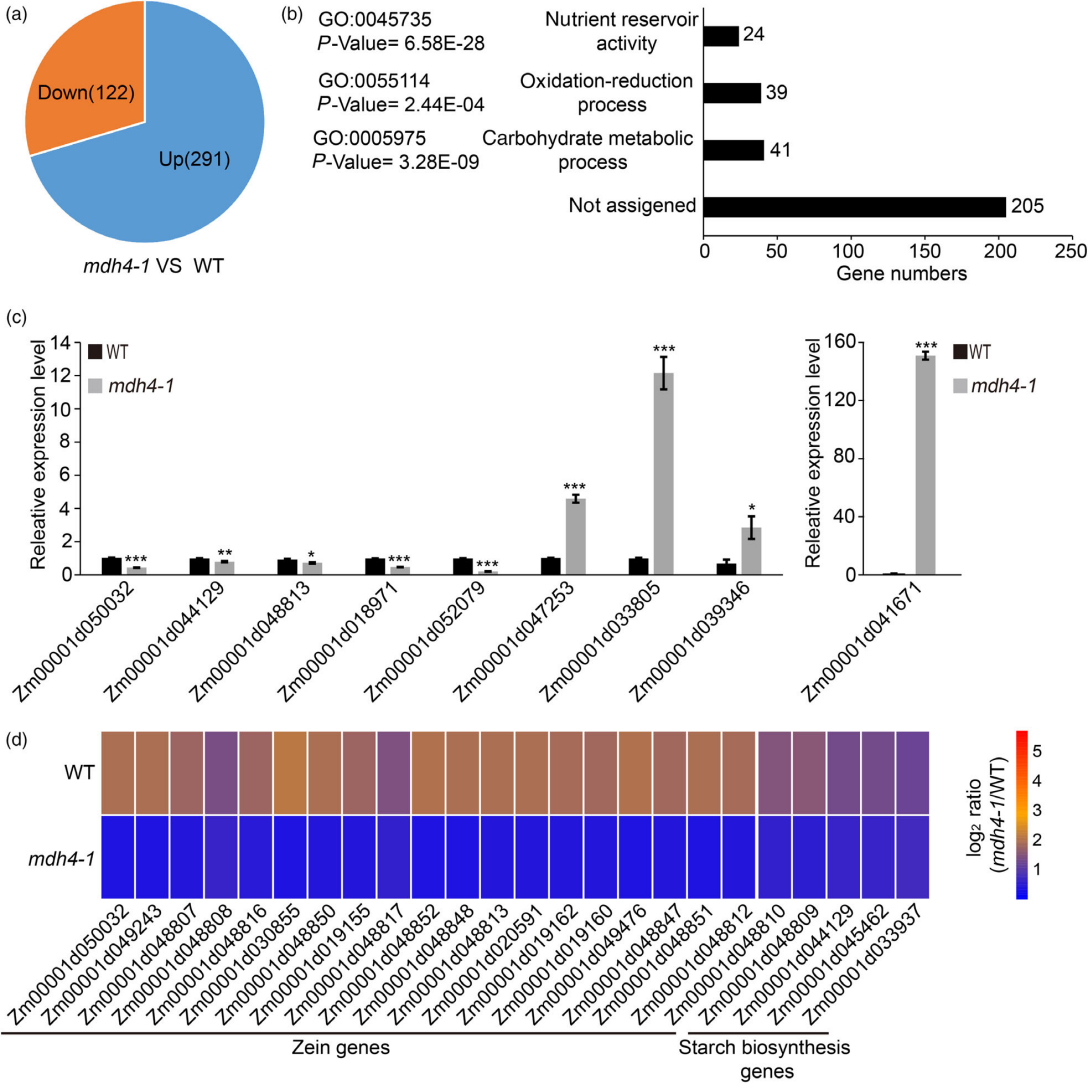
Transcriptomic analysis of WT and *mdh4‐1* kernels. (a) The numbers of differentially expressed genes in *mdh4‐1* compared with WT. (b) The GO terms of functionally annotated DEGs. (c) The selected genes were verified by qPCR with three replicates. Values are represented as means ± SE, ***P < 0.001 (Student's *t‐*test). (d) Heat map showing differentially expressed genes implicated in Zein and starch biosynthesis.

To further explore the molecular basis of the metabolic changes in the *mdh4‐1* mutant, 12 DAP mutant and WT kernels were subjected to RNA‐seq analysis. A total of 23 594 transcripts were detected and retrieved, among which 413 were differentially expressed [log2 (fold change) > 0.78 or <−1 and false discovery rate (FDR) < 0.01] between the WT and *the mdh4‐1* mutant. Of the differentially expressed genes (DEGs), 291 were up‐regulated and 122 were down‐regulated in the *mdh4‐1* mutant as compared to WT (Figure 8a). Of those, 309 DEGs could be functionally annotated by BLAST searches against the UniProt database (http://www.uniprot.org) and were analysed by an online Gene Ontology (GO) software (agriGO, Tian *et al*., 2017). The significantly enriched terms included carbohydrate metabolic process (GO: 0005975), oxidation‐reduction process (GO: 0055114) and nutrient reservoir activity (GO: 0045735) (Figure 8b; Table S7).

Several genes were randomly selected and tested with quantitative real‐time PCR (qPCR) to validate the RNA‐seq results. Consistent with the RNA‐seq data, the transcript levels of Zm00001d018971 (*O2*), Zm00001d044129 (*Sh2*), Zm00001d048813 (*zein‐alfa*), Zm00001d050032 (*Bt2*) and Zm00001d052079 (*LKR/SDH*) were lower, and those of Zm00001d033805, Zm00001d039346, Zm00001d041671 (*BETL3*) and Zm00001d047253 (*SuSy*) were higher in *mdh4‐1* than in WT (Figure 8c; Table S7). DEGs under carbohydrate metabolic processes (GO: 0005975) and starch biosynthesis and metabolic processes (GO: 0019252 and GO: 0005982) were enriched (Table S7). Key genes involved in starch and amylose biosynthesis, including Zm00001d044129 (*Sh2*), Zm00001d050032 (*Bt2*), Zm00001d033937 (*GBSSI*) and Zm00001d045462 (*wx1*), were significantly down‐regulated in the *mdh4‐1* mutant. DEGs under nutrient reservoir activity (GO: 0045735) were found to be mainly involved in zein protein biosynthesis (21/24). These DEGs were also significantly down‐regulated the *mdh4‐1* mutant (Figure 8d; Table S7). The changed expression levels of genes related to starch and protein biosynthesis suggest a role of *ZmMdh4* in storage reserve accumulation.

## Discussion

### *ZmMdh4* is indispensable for kernel development

Gooman and associates have reported that the kernels of homozygous *Zmmdh4* and *Zmmdh5* mutants were viable and could germinate and develop to normal mature plants (Goodman *et al*., 1981). In this study, both the natural *mdh4‐1* mutant and the cas9 *mdh4‐2* and *mdh4‐3* lines could develop into relatively normal plants but exhibited poor seed germination, retarded vegetative and reproductive growth compared with controls (Figures 1, 4a‐b; S4). It is worth noting that *ZmMhd5*, the only paralog of *ZmMhd4*, is unlinked to the mutant kernel phenotype (Figure 4e‐h). Our findings point to an indispensable role for *ZmMdh4* in kernel and plant development. Previous studies have reported a positive correlation between the transcript level and enzymatic activity of cytosolic MDHs in apple and cotton (Imran *et al*., 2017; Yao *et al*., 2011b). Taken together with the lack of obvious morphological and developmental defects in the *mdh4‐1* and *mdh4‐cas9* lines, these results indicate the existence of other genetic factors that are functionally redundant to *ZmMdh4*.

### ZmMDH4 is indispensable for ATP production

In non‐photosynthetic organs, such as the endosperm, glycolysis in cytoplasm and the TCA cycle in mitochondria are major sources for ATP. The cytosolic MDHs, which catalyse the reversible conversion between malate and OAA, contribute to the partitioning of metabolic flux between glycolysis and the TCA cycle (Selinski and Scheibe, 2019), thereby regulate ATP production. Consistent with this function, the loss of ZmMDH4 activity caused substantial changes in the levels of glycolysis and TCA cycle‐related metabolites in *mdh4‐1* (Figure 7a‐b and h). For example, an increase in lactate level was observed, as well as a reduction in ATP content in *mdh4‐1* compared with the WT (Figure 7b, d), implying enhanced glycolysis and a role of a malate/OAA shuttle in regulating ATP production (Scheibe, 2004). As a result, the mitochondria of *mdh4‐1* were vacuolated, NADH oxidase activity was reduced, and the mitochondrial I + III super‐complex was disassociated (Figure 7c‐g). These data are in line with a previous report that the rice *flo16* mutant, which was determined to be caused by a mutated cytosolic MDH, had reduced ATP production (Teng *et al*., 2019). These data suggest that cytosolic ZmMDH maintains mitochondrial complex activity, ATP production and the homeostasis of glycolysis and the TCA cycle.

### ZmMDH4 affects starch and zein synthesis in the endosperm

In the cytoplasm, pyruvate phosphate dikinase (PPDK), which reversibly converts PEP to pyruvate, also involved in the malate metabolic pathway. Knockout of maize endosperm PPDK (cytosolic *pdk2*) results in *opaque* kernel characteristics, elevated glycolysis metabolites, reduced ATP content, but unaffected starch and zein contents (Lappe *et al*., 2018). However, mutations in *ZmMdh4* cause an elevation in glycolysis metabolites and a reduction in ATP content, with a concomitant decrease in starch and zein contents (Figures 1d‐e, 7a‐d). These results suggest that different regulators might be involved in cytosolic *pdk2* and *ZmMdh4* expression to balance glycolysis and the TCA cycle, as indicated that PPDK is the direct target of O2 and MDH is the target of thioredoxin‐h1 (Hara *et al*., 2006; Lappe *et al*., 2018; Manicacci *et al*., 2009). Loss‐function of the cytosolic *Mdh* leads to increased oxidation, which subsequently results in a reductive ADP‐glucose pyrophosphorylase (AGP, the committed enzyme of starch biosynthesis) activation state, thereby reducing starch content in tomato plastids (Centeno *et al*., 2011) and in rice endosperm (Teng *et al*., 2019). These findings are consistent with increased NAD^+^/NADH levels, as well as the reduced expression levels of *Bt2* and *Sh2*, which encode the large and small subunits of AGP observed in the *mdh4‐1* (Figures 7c and 8c). Thus, it can be inferred that restricted AGP activity causes reduced starch accumulation in *mdh4‐1* mutants. Another reason could be related to compromised ATP production, which would directly impair the differentiation of BETL cells, or the activity of endosperm cells (Figure 2b‐c). This would inhibit the biosynthesis and deposition of storage compounds, resulting in small kernels.

Zeins are the most abundant seed storage proteins (>60%; Wu and Messing, 2014) that determine the nutritional quality of maize grain (Frizzi *et al*., 2010; Hunter *et al*., 2002). Published data have demonstrated that mutants with reduced zein contents, especially α‐zein, such as the *o2*, *o7*, *ocd1* and *fl2* mutants, accumulate lysine, an essential amino acid whose levels in maize grains is not well balanced for human and animal consumption, to compensate (Coleman *et al*., 1997; Kemper *et al*., 1999; Miclaus *et al*., 2011; Wang *et al*., 2011; Yang *et al*., 2018). Analogous to that observed with these mutants, the biosynthesis of zein (especially 19‐ and 22‐kD α‐zein) was restricted in the *mdh4‐1* mutant and the lysine content was increased in the *mdh4‐1* mutant; this was further supported by the down‐regulation of all DEGs involved in zein biosynthesis and lysine degradation, and enhanced levels of aspartate for lysine and glutamine biosynthesis, which competes with glutamate for zein biosynthesis in the *mdh4‐1* (Figures 1e‐f, 8c‐d). Collectively, these results demonstrate that *ZmMdh4* influences TCA‐derived substrate supply for protein biosynthesis, though the gap between ZmMDH4 activity and the final seed phenotype needs to be comprehensively investigated.

**Figure 9:**
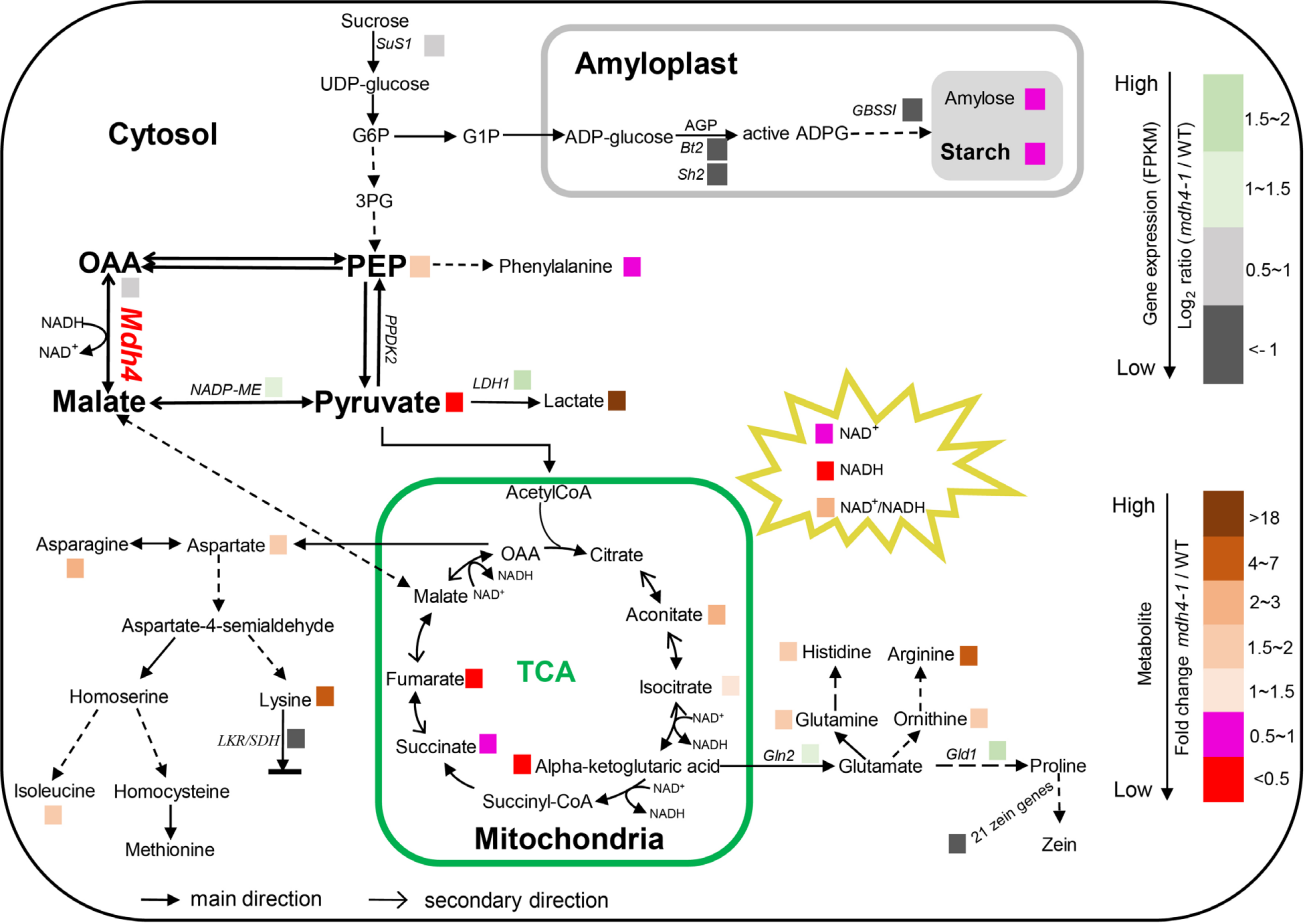
A proposed regulatory model of *ZmMdh4* in maize endosperm development. *SuS1*, sucrose synthase1; *Bt2*, brittle2; *Sh2*, shrunken‐2; *GBSSI*, granule‐bound starch synthase I; *Mdh4,* malate dehydrogenase 4; *NADP‐ME*, NADP malic enzyme; *LDH1*, lactate dehydrogenase1; *LKR/SDH,* lysine‐ketoglutarate reductase/saccharopine dehydrogenase; *Gln2*, glutamine synthetase 2; *Gld1*, glutamate decarboxylase 1; *PPDK2*, pyruvate phosphate dikinase; G6P, glucose‐6‐phosphate; G1P, glucose‐1‐phosphate; 3PG, 3‐phosphoglycerate; OAA, oxaloacetate; PEP, phosphoenolpyruvate; AGP, ADP‐glucose pyrophosphorylase.

In summary, we cloned the *ZmMdh4* gene that encodes cytosolic malate dehydrogenase in maize. The 3‐bp deletion in exon 7 eliminates ZmMDH4 enzymatic activity, resulting in reduced ATP supply and an elevated oxidation level, perturbing AGP activity and starch production in the endosperm. Concomitantly, impaired TCA cycle alters the substrate availability for amino acid biosynthesis, which influences the proportions of zein and non‐zein protein by modulating the expression levels of related genes in the endosperm as depicted in our proposed model (Figure 9).

## Methods

### Plant materials

*mdh4‐1* is a naturally occurring mutant isolated during maize breeding and was crossed with Zheng58, PH6WC, HCL645, Qi319, Lx9801 and D1798Z to generate F_1_ hybrids. The resulting F_1_ plants were self‐pollinated to construct six F_2_ mapping populations. The *mdh4* mutant, Zheng58, PH6WC, HCL645, Qi319, Lx9801, D1798Z and the F_2_ populations were grown at the research farm of Henan Agricultural University, Zhengzhou, China (113°42′ E, 34°48′ N). The near‐isogenic lines (NILs) of *mdh4‐1* were produced by self‐crossing heterozygous F_2_ individuals, derived from the by crossing Zheng58 with *mdh4‐1*, for eight generations under background and foreground marker‐assisted selection. The ears and kernels of the F_2_ plants and NILs were collected from no less than three individuals at 5, 8, 10, 11, 12, 14, 16, 20, 23, 25, 27, 30 and 35 DAP.

### Cytological section preparation

To prepare the paraffin sections of kernels, immature seeds were fixed overnight at 4 °C in a formalin‐acetic acid‐alcohol (FAA) solution containing 50% ethanol, 5% acetic acid and 3.7% formaldehyde. The fixed materials were then dehydrated in an ethanol gradient series (50, 70, 85, 95 and 100% ethanol). Afterwards, the samples were treated with xylene, embedded in paraffin wax via infiltration and cut into 6–10 µm‐thick sections under Leica RM2235 (Germany). The sections were stained with toluidine blue (Sinopharm Chemical Reagent Co., Ltd) and examined under the Lecia M165FC stereomicroscope (Germany). The endosperm structures of mature and immature seeds of different developmental stages were observed by scanning electron microscopy (SEM) and transmission electron microscopy (TEM), respectively (Wu and Messing, 2010; Zhang *et al*., 2016).

### Map‐based cloning of *ZmMdh4*

A total of 34 080 F_2_ individuals were used for map‐based cloning. The genotypes of key F_2_ recombinants were verified by examining the corresponding F_3_ seeds from each F_2_ ear. Table S3 lists the primers used. Additional polymorphic markers were developed by comparing genomic sequence near the *ZmMdh4* locus between *mdh4‐1* and Zheng58.

### Vector construction and gene transformation

To construct the CRISPR/Cas9 vector, the recombinant PBUE411 vector was designed to produce mutations within the coding region of *ZmMdh4*, using a small guide RNA (sgRNA) alongside the *Cas9* endonuclease gene. To obtain *ZmMdh4* overexpression lines (*Mdh4‐OE*), the CDS driven by a Ubi promoter was cloned into a CUB skeleton vector. The CRISPR/Cas9 and overexpression constructs were introduced into *agrobacterium* strain EHA105 and used to transform the immature embryos of maize inbred line ZZC01, by co‐cultivation at the Life Science and Technology Center of China National Seed Group Co. Ltd (Wuhan, China). Positive transgenic plants were confirmed by amplifying the *Bar* gene and the knock‐out regions by PCR using *ZmMdh4‐*specific primers CUB‐F/CUB‐R, which span *ZmMdh4* and the CUB vector. A co‐dominant functional marker for the 3‐bp InDel in exon 7, Exon7‐L/Exon7‐R, was developed to identify the homozygous *mdh4‐1* genotype. All primers used for vector construction and gene transformation are listed in Table S3.

### Starch, protein and total amino acid determination

A minimum of 20 endosperms from mature kernels of the WT and *mdh4‐1* mutant were pulverized into fine powder using a pulverizer. For each sample, 50 mg flour was used to measure starch content using the Megazyme kit (K‐TSTA; Megazyme). SDS‐PAGE was used to analyse the accumulation patterns of zein and non‐zein proteins in both the WT and *mdh4‐1* mutant following previously published procedures (Liu *et al*., 2016; Zhang *et al*., 2016). Total amino acids (free amino acids and protein‐bound amino acids) in the mature kernels were analysed according to the method of Wang *et al*. (2011) with three replicates.

### RNA extraction and quantitative real‐time PCR (qPCR)

Total RNA was extracted from immature endosperms, embryos and other tissues using TransZol Plant (Transgen). Five hundred nanogram of total RNA was used for first‐strand cDNA synthesis using HiScript® QRT SuperMix for qPCR (+gDNA wiper) (Vazyme). All qPCR analyses were carried out in a Bio‐Rad iQ5 system (Bio‐Rad iQ5 Real Time PCR, ABI 7500) using the SYBR Green I kit (Vazyme). The 2^−ΔΔCT^ method was used to calculate the relative transcript level of the target gene with *ZmActin* (Zm00001d010159) as the endogenous control. The PCR program was conducted as follows: (1) 5 min at 94 °C; (2) 40 cycles of 10 s at 95 °C, 30 s at 58 °C. The 20‐μL reaction volumes contained 2 μL cDNA, 0.4 μL L/R primers (10 μm), 10 μL 2 × qPCR SYBR Green I mix and 7.2 μL double‐distilled water. Statistically significant differences in gene expression levels were analysed by Student's *t*‐test. All primers used for qPCR are listed in Table S3.

### Subcellular localization of ZmMDH4

The CDS of *ZmMdh4* was fused with the enhanced GFP (eGFP) reporter gene and cloned into the pCAMBIA1300 vector (35S::ZmMDH4:GFP). pCAMBIA1300‐35S‐GFP vector not containing the *ZmMdh4* gene was used as the free GFP control (35S::GFP). The 35S::GFP and 35S::ZmMDH4:GFP constructs were transiently expressed in *Nicotiana benthamiana* (*N. benthamiana*) leaves and Arabidopsis mesophyll protoplasts as described by Li *et al*. (2017) and Yang *et al*. (2018). GFP signal was observed and imaged using a confocal microscope (FV1000, Olympus). All primers used for the subcellular localization analysis are listed in Table S3.

### Protein extraction and Western blot

Endosperm proteins were isolated using the method described by Wu and Messing (2012), separated on a 15% SDS PAGE by electrophoresis, and transferred to a polyvinylidene difluoride (PVDF) membrane (Bio‐Rad). The membrane was then incubated with commercial MDH4 (AS153065, Agrisera) and ACTIN (Abclonal, China) antibodies and visualized using the Tanon‐5200 system (Tanon Science & Technology Co., Ltd.). The MDH4 and ACTIN antibodies were 1:2000 and 1:5000 diluted, respectively (Yang *et al*., 2018). The NAD7 (PHY1077S, Phytoab), SDH1 (PHY0558S, Phytoab) and CYC1 (PHY0566S, Phytoab) antibodies were 1:3,000 diluted, while the α‐tubulin antibody (AS10680, Agrisera) was 1:10 000 diluted.

### Enzymatic assay of ZmMDH4

The open reading frames (ORFs) of *ZmMdh4* and *Zmmdh4* were amplified by PCR with gene‐specific primers (Table S3) and separately cloned into the pET‐28a vector using a One Step Cloning Kit (Vazyme). The resulting *ZmMdh4* and *Zmmdh4* constructs were each transformed into the *Escherichia coli* (*E. coli*) strain BL21 (DE3). 2 mL of the transformed cells was used to inoculate 200 mL LB media in a 500 mL conical flask and cultured at 37 °C until OD_600_ reached 0.4–0.6, when 0.5 mm isopropyl‐β‐d‐thiogalactoside (IPTG) was added to induce protein expression. After culturing at 16 °C overnight, the cells were collected by centrifugation and then resuspended in 10 mL lysis buffer [50 mm Tris‐HCl pH 8.0, 50 mm NaCl, 5% glycerol, and 5 mm imidazole, 0.1 mm phenylmethanesulfonyl fluoride (PMSF) and 0.0037% β‐mercaptoethanol]. Next, the cells were disrupted by sonicating for 30 min and centrifuged at 5752 *g* for 50 min. The supernatant was added to the Ni‐NTA resin, which was pre‐equilibrated with 10 mL Lysis buffer, and incubated with 6 mL lysis buffer to rinse the resin. Finally, the proteins were eluted by a series of elution buffers (Lysis buffer + imidazole) containing 10 mm, 50 mm, 100 mm and 200 mm imidazole. The eluted protein fractions were pooled and dialysed with a 10 kDa ultrafiltration device to remove imidazole and salt, evaluated for purity via SDS‐PAGE electrophoresis, and purified by dialysis buffer (50 mm Tris‐HCl pH 8.0, 20 mm NaCl, 5% glycerol and 0.037% β‐mercaptoethanol). The purified proteins were quantified using a calibration curve based on the absorbance at 280 nm (A_280_) with BSA as a standard.

The 1 mL oxidative/reductive catalytic reaction included 250 mm HEPES buffer (PH 8.0), 2 mm MgCl_2_, 0.25 mm NAD^+^/NADH, 2.5 mm OAA or malate (Solarbio, Beijing, China), and 5 μg (1 μg/μL) of each protein sample. The change in NADH concentration (A_340_) of each reaction was monitored for 6 min by a spectrophotometer (DU^®^730, Nucleic Acid/Protein Analyzer, BeckMan) at 1 min intervals.

### Determination of amino acids and energy metabolites

12‐DAP kernels of the WT and *mdh4‐1* mutant were collected with three biological replications, snap frozen in liquid nitrogen, and stored at −80 °C until use. More than 3 g of kernels were ground and 55 mg fine powder from each sample was mixed with 1 mL of pre‐cooled methanol/acetonitrile/H_2_O solution and vortexed for 30 s. The mixture was then sonicated for 30 min on ice and left at −20 °C for 1 h to allow protein precipitation. Afterwards, the mixture was centrifuged for 15 min at 14 000 ***g*** at 4 °C, and the proteins were vacuum dried using a lyophilizer (FD‐1D‐80, BILON, Shanghai). The dried protein extracts were then dissolved in 100 μL 1:1 (v:v) mixture of acetonitrile:H_2_O and centrifuged at 14 000 ***g*** at 4 °C for 15 min. Targeted metabolic analysis was performed using the LC‐MS/MS system at Shanghai Applied Protein Technology Co. Ltd. Electrospray ionization was conducted with an Agilent 1290 Infinity chromatography system and AB SCIEX QTRAP 5500 mass spectrometer.

ATP content was determined using an ATP Assay Kit (Beyotime) following the manufacturer's instructions; three biological replicates were analysed for each sample. Briefly, 100 mg fresh endosperm of WT and *mdh4‐1* was homogenized in 1 mL pre‐cooled lysis buffer and centrifuged at 12 000 ***g*** for 5 min at 4 °C. The supernatant was used to determine ATP content. ATP standard solutions of various concentrations (0, 0.025, 0.05, 0.1, 0.2, 0.5, 1 µm) were prepared to generate an ATP calibration curve. The supernatant and ATP standards were separately mixed with the ATP detection solution (working concentration) provided in the Kit in a 1:9 ratio. Luminescence was detected by a Tecan Spark 20M microplate reader (Shanghai), and the ATP content of each sample was calculated based on the calibration curve.

### BN‐PAGE and the determination of mitochondrial complex activity

The mitochondria were separated from 15 DAP kernels using the Plant mitochondria DNA Extraction Kit (Beijing biolab technology co. LTD) with minor modifications. Briefly, about 400mg kernel was ground in liquid nitrogen and 1.6 mL plant cell lysis buffer (0.5% β‐mercaptoethanol) was added to each sample. The samples were mixed and centrifuged at 1000 ***g*** for 5 min at 4 °C. Then, the supernatant was transferred to a new tube and centrifuged at 16 000 ***g*** for 10 min at 4 °C. Crude mitochondria were resuspended in cleanout fluid and centrifuged at 1000 ***g*** for 5 min at 4 °C. The resulting supernatant was centrifuged at 16 000 ***g*** for 10 min at 4 °C to collect highly purified mitochondria and the pellet was resuspended in 100 µL B25G20 solution. BN‐PAGE and in‐gel activity assay of mitochondrial complexes were performed as described by Chen *et al*. (2017).

### RNA‐seq analysis

Total RNA was extracted from 12 DAP endosperms of the *mdh4‐1* mutant and WT with RNAprep Pure Plant Kit (Tiangen). The VAHTSTM Stranded mRNA‐seq Library Prep Kit for Illumina® (Vazyme) was used to construct the RNA‐seq libraries. Clean reads were obtained using the Illumina HiSeq X Ten platform (JiakangBio, Wuhan, China) and mapped to the B73 reference genome (RefGen_V4) using Bowtie2. Gene expression level was converted to Fragments per Kilobase Million (FPKM) for each transcript model. Differentially expressed genes (DEGs) were selected by the following criteria: log_2_(fold change) > 0.78 or <1, false‐discovery rate (FDR) <0.05, as calculated by the DEseq2 software, and *P*‐value < 0.05. GO enrichment analysis of the DEGs was performed using an online version of agriGO (Tian *et al*., 2017).

## Conflict of interest

This study did not involve human participants and/or animals. All authors declare no financial or commercial conflicts of interest.

## Author contributions

Z.F. and J.T. designed and supervised this study. Y.C., H.Z., R.T., H.Y., C.S., L.W. and W.Z. performed the experiments. Z.F., Y.C., Z.G. and X.Z performed the data analysis. Z.F., Y.C. and J.T. prepared the manuscript with inputs from other authors.

## Supporting information


**Figure S1** Dynamic development of the kernels on F_2_ ears. The red arrows indicate mutant kernels.
**Figure S2** Phenotype of *mdh4‐1* kernels and kernel segregation in other genetic backgrounds.
**Figure S3** Multisequence alignment showing the distribution of the 3‐bp Indel in teosinte and 55 diverse maize inbred lines.
**Figure S4** Phenotypic characteristics of transgenic lines.

**Table S1** Field evaluation of normal and *mdh4‐1* kernel segregation in different F_2_ populations.
**Table S2** Amino acid level determination in mature WT and *mdh4‐1* kernels.
**Table S3** Primers used in this study.
**Table S4** *ZmMdh4* polymorphisms identified in the association panel.
**Table S5** Association analysis of *ZmMdh4*.
**Table S6** Allelism test using heterozygous *mdh4‐1* and homozygous *mdh4‐2 and mdh4‐3* T_3_ plants.
**Table S7** Gene ontology classifications of DEGs with functional annotation.
