# Supplementary material for: Cytosolic malate dehydrogenase 4 modulates cellular energetics and storage reserve accumulation in maize endosperm
Source: Plant Biotechnol J. 2020 Jun 14;18(12):2420–35. doi: 10.1111/pbi.13416 (PMC7680550; doi:10.1111/pbi.13416)
Supplement: Supplementary file 1 — Figure S1 Dynamic development of the kernels on F2 ears. The red arrows indicate mutant kernels. Figure S2 Phenotype of mdh4‐1 kernels and kernel segregation in other genetic backgrounds. Figure S3 Multisequence alignment showing the distribution of the 3‐bp Indel in teosinte and 55 diverse maize inbred lines. Figure S4 Phenotypic characteristics of transgenic lines. [file PBI-18-2420-s001.docx]

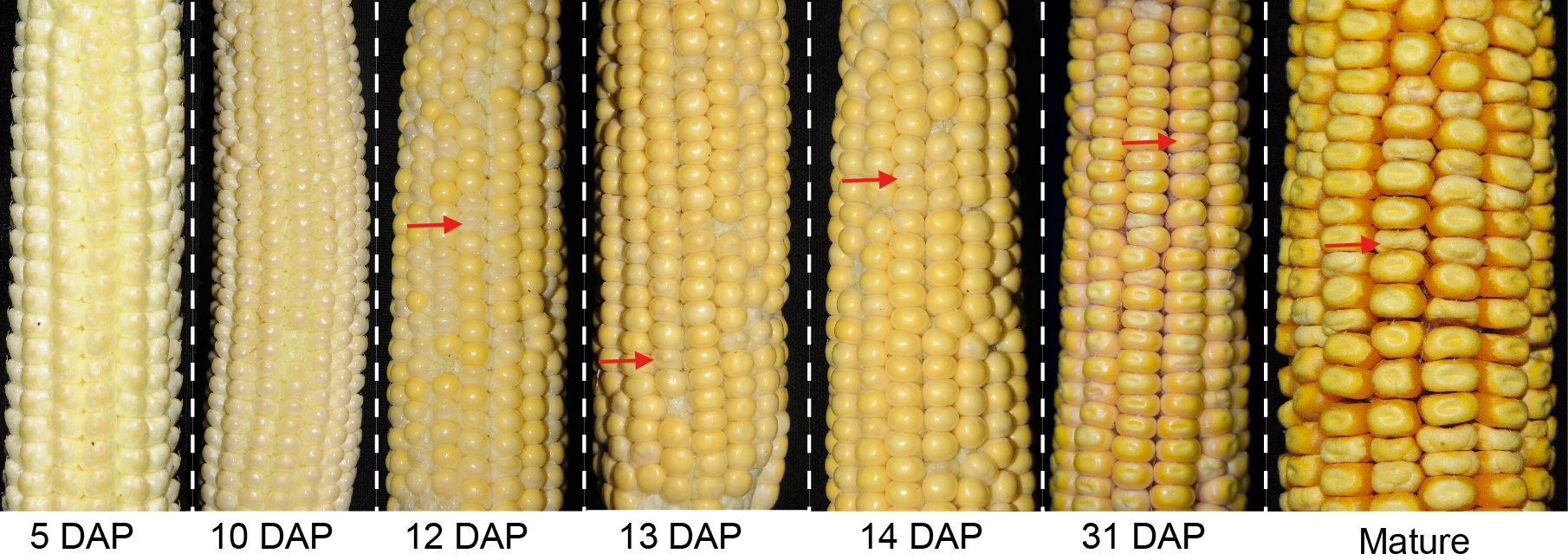


**Figure S1.** Dynamic development of the kernels on F_2_ ears. The red arrows indicate mutant kernels.
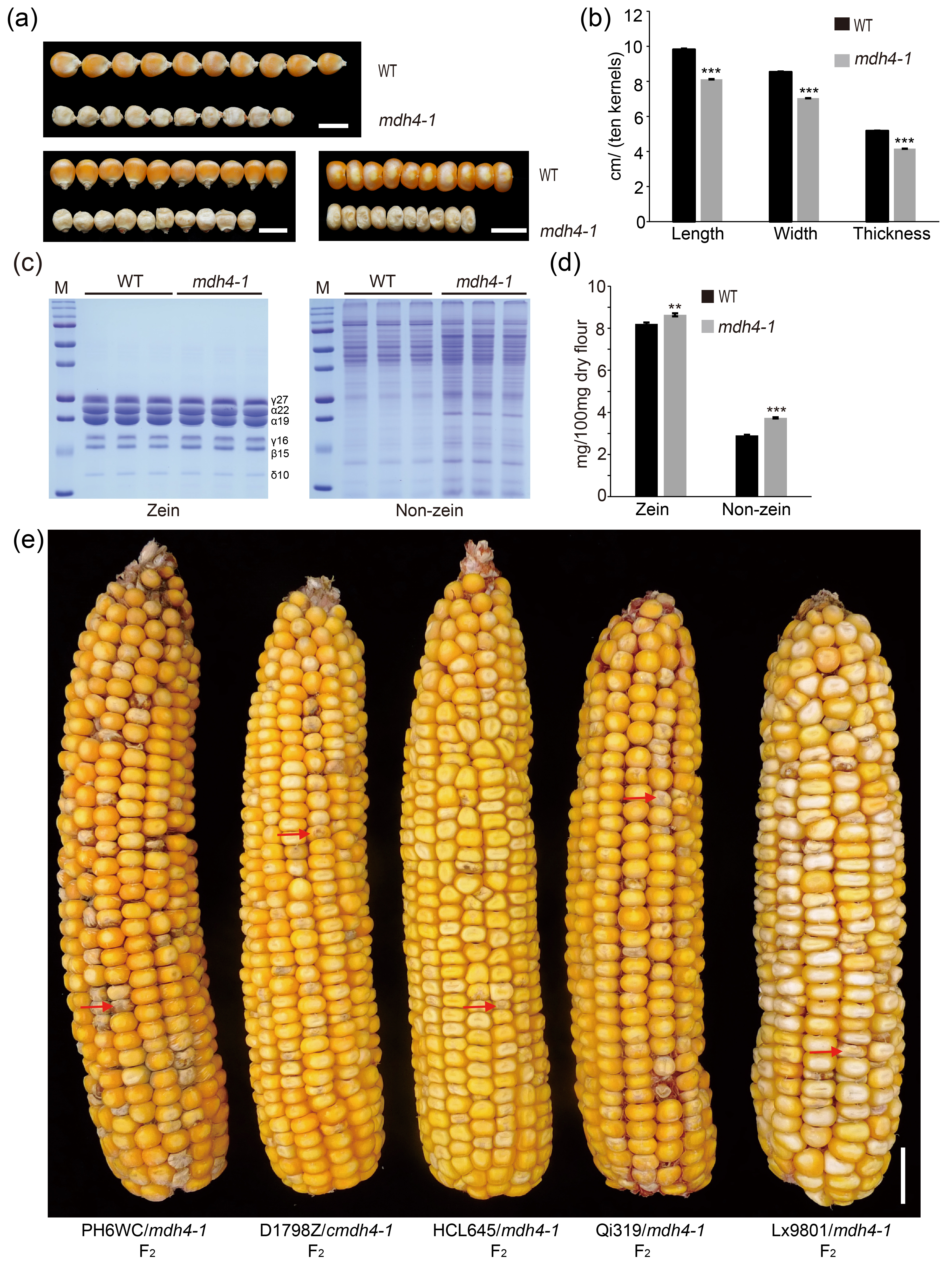


**Figure S2.** Phenotype of *mdh4-1* kernels and kernel segregation in other genetic backgrounds. (a) Representative WT and *mdh4-1* kernels; scale bar, 1 cm. (b) Comparison of kernel length, width and thickness between WT and *mdh4-1*. (c) SDS-PAGE analysis of zein and non-zein protein in mature WT *vs*. *mdh4-1* kernels. M, protein standard (d) Analysis of storage reserve accumulation in mature WT and *mdh4-1* kernels. Values are represented as means ± SE, ^*^*p* < 0.05, ^***^*p* < 0.001 (Student’s t-test). (e) Representative ears from *mdh4-1* mutants in different genetic backgrounds, scale bar; 3 cm. The red arrows indicate mutant kernels. The F_2_ ears generated by PH6WC × *mdh4-1*, D1798Z × *mdh4-1*, HCL645 × *mdh4-1*, Qi319 × *mdh4-1*, and Lx9801 × *mdh4-1*.


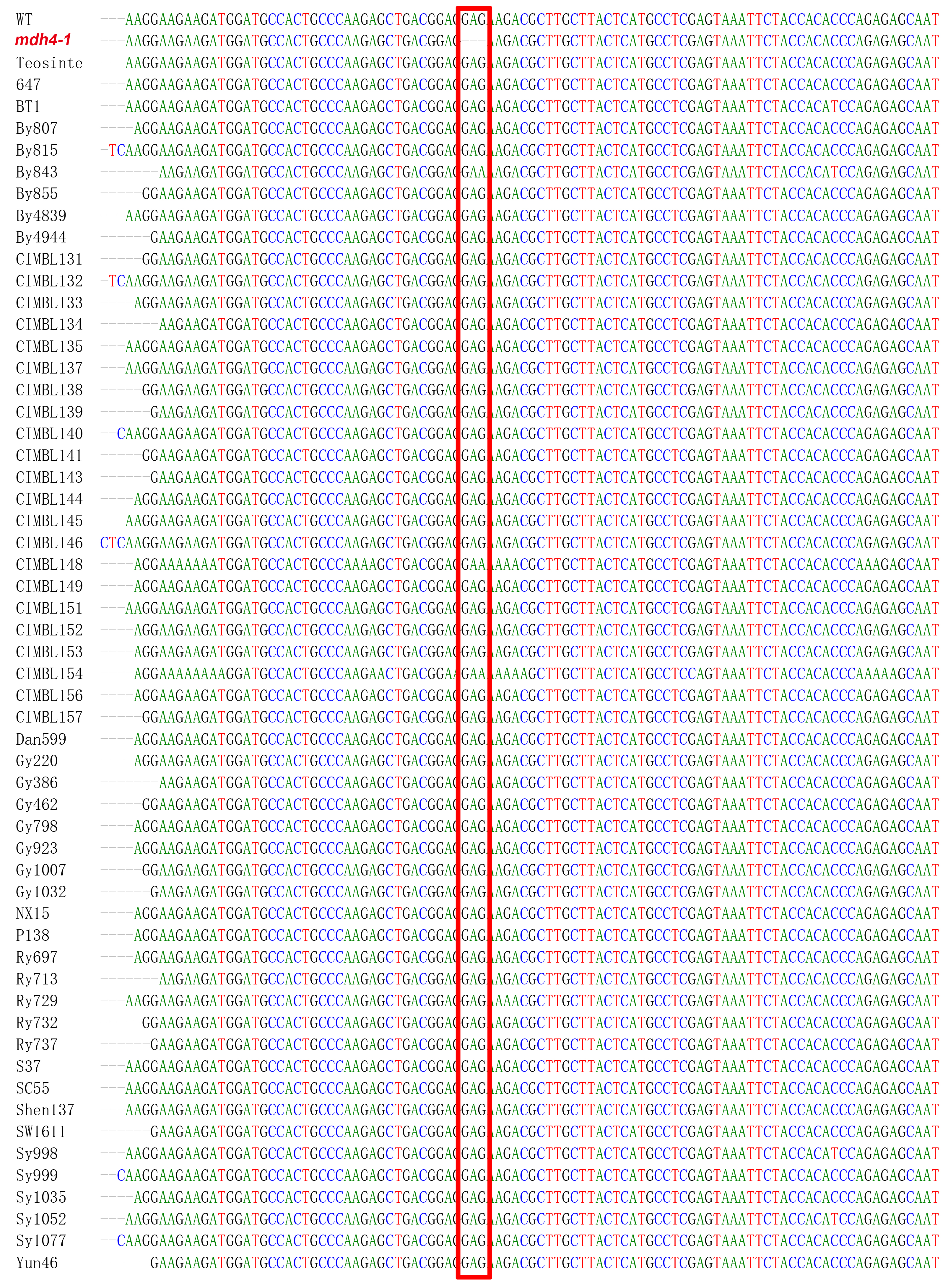


**Figure S3.** Multisequence alignment showing the distribution of the 3-bp Indel in teosinte and 55 diverse maize inbred lines.


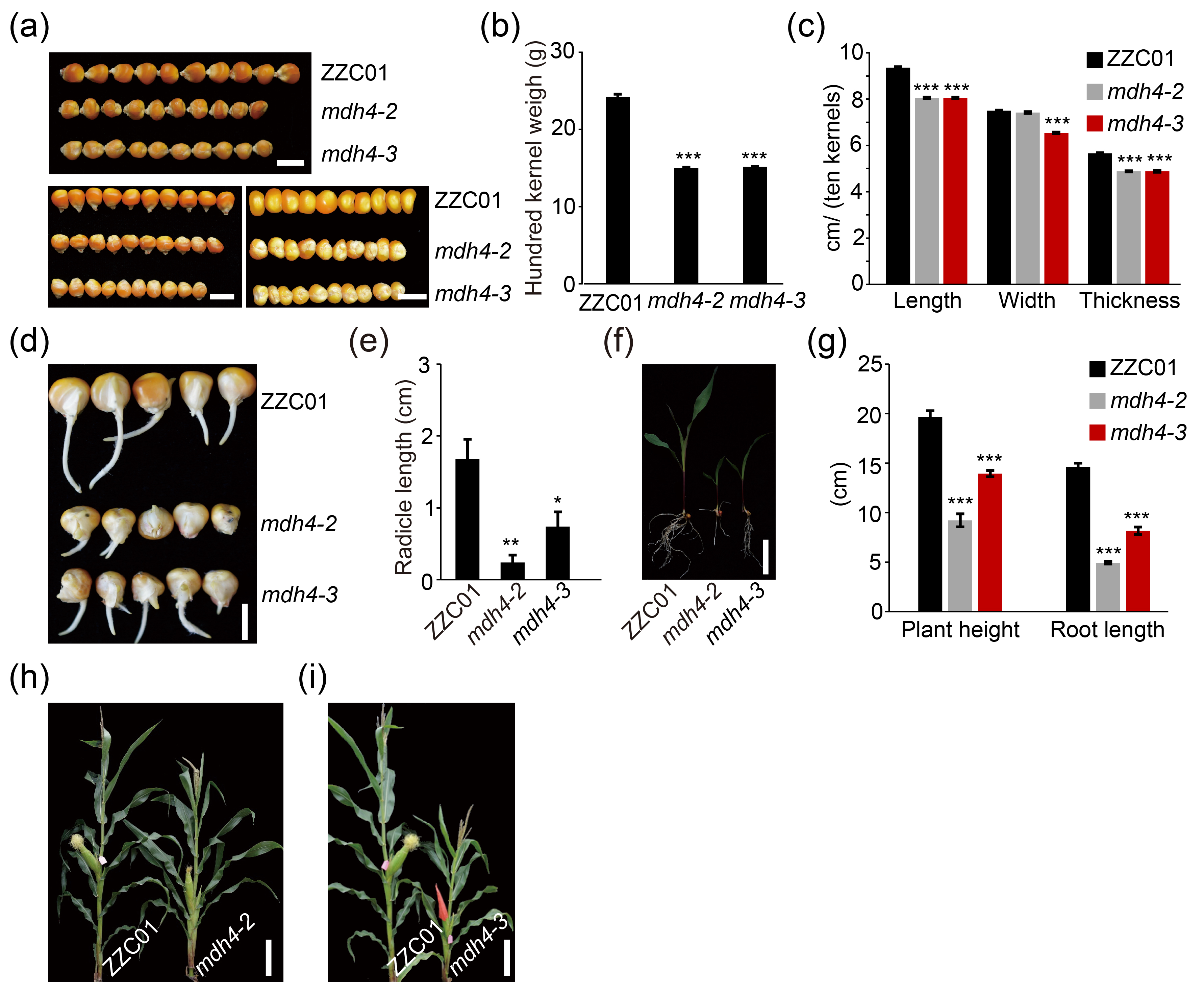


**Figure S4.** Phenotypic characteristics of transgenic lines. (a) Representative kernels from ZZC01, *mdh4-2* and *mdh4-3*; scale bar, 1 cm. (b) 100-kernel weight between *mdh4-2*, *mdh4-3*, and ZZC01, with five replicates. (c) Comparison of kernel length, width and thickness between ZZC01, *mdh4-2* and *mdh4-3.* (d) Representative germination of ZZC01, *mdh4-2* and *mdh4-3*; scale bar, 5 cm. (e) Comparison of radical length between ZZC01, *mdh4-2* and *mdh4-3*; scale bar, 1 cm*.* (f) Representative seedings of ZZC01, *mdh4-2* and *mdh4-3* 13 days after germination (DAG); scale bar, 5 cm. (g) Plant height and root length of *mdh4-2*, *mdh4-3* and ZZC01 seedlings. (h-i) Representative mature plants from ZZC01, *mdh4-2* and *mdh4-3*; scale bars, 20 cm. All bar graphs, values are represented as means ± SE, ^*^*p* < 0.05, ^**^*P* < 0.01, ^***^*p* < 0.001 (Student’s t-test).

**Table S1.** Field evaluation of normal and *mdh4-1* kernel segregation in different F_2_ populations.

**Table S2.** Amino acid level determination in mature WT and *mdh4-1* kernels.

**Table S3.** Primers used in this study.

**Table S4.** *ZmMdh4* polymorphisms identified in the association panel.

**Table S5.** Association analysis of *ZmMdh4*.

**Table S6.** Allelism test using heterozygous *mdh4-1* and homozygous *mdh4-2 and mdh4-3* T_3_ plants.

**Table S7.** Gene ontology classifications of DEGs with functional annotation.
